# Traditional Uses, Phytochemistry, Pharmacology, and Toxicology of *Belamcanda chinensis*: A Review

**DOI:** 10.3390/plants14233688

**Published:** 2025-12-03

**Authors:** Tieqiang Zong, Mingxia Li, Zhengyu Hu, Long Jin, Yanan Liu, Yuanqi Duan, Jinfeng Sun, Wei Zhou, Gao Li

**Affiliations:** Key Laboratory of Natural Medicines of the Changbai Mountain, Ministry of Education, College of Pharmacy, Yanbian University, Yanji 133000, China; 0000008134@ybu.edu.cn (T.Z.); 17808005570@163.com (M.L.); 0000007948@ybu.edu.cn (Z.H.); longjin0209@163.com (L.J.); 18243390807@163.com (Y.L.); duanyuanqi163@163.com (Y.D.)

**Keywords:** *Belamcanda chinensis* (L.) Redouté, traditional uses, phytochemistry, pharmacology activities, toxicity, clinical application

## Abstract

The *Belamcanda chinensis* (L.) Redouté is a perennial herb belong to the genus *Belamcanda*, primarily found in China, but with additional distribution in North Korea, South Korea, Japan, and India. The rhizomes of *B. chinensis* have a long history of use as a traditional herbal medicine in China, one that is recognized for its effects in clearing heat, in detoxifying and eliminating phlegm, and in soothing the throat. In this review, we conducted a comprehensive search across several databases, both Chinese and international, using the primary keyword *Belamcanda chinensis* paired with a relevant research area (e.g., chemical composition, pharmacology). The databases included Sci-Finder, ScienceDirect, PubMed, China National Knowledge Infrastructure, Wiley, Springer Baidu Scholar and Research Gate, as well as domestic materia medica. We illustrated the chemical structures using ChemBioDraw Ultra 22.0 software. There are more than 10 proprietary Chinese medicines already on the market that consist of or originate from *B. chinensis*. More than 200 natural products have been isolated and identified from *B. chinensis*, including iridal-type triterpenoids, flavonoids, phenolics, quinones, sesquiterpenes, and polysaccharides. Modern pharmacological studies indicate that both crude extracts and monomeric compounds exhibit anti-inflammatory, anti-tumor, antioxidant, neuroprotective and anti-diabetic activities, with potential regulatory pathways. Additionally, *B. chinensis* demonstrates toxicity to fish, mollusks and arthropods. Clinical studies have shown that formulas containing *B. chinensis* as the main ingredient have a good therapeutic effect on respiratory diseases. In summary, *B. chinensis* presents promising prospects for application in medicine, functional food, cosmetics and agriculture. Therefore, we have reviewed the chemical composition, pharmacological activities (both in vivo and in vitro), structure–activity relationships, toxicity and clinical application of *B. chinensis* over the past 40 years, aiming to provide a theoretical basis for the subsequent comprehensive utilization of the plants.

## 1. Introduction

*Belamcanda chinensis* (L.) Redouté is a perennial herb belonging to the Iridaceae family and is widely distributed across Northeast Asia, including regions such as China, North Korea, South Korea and Japan, as well as in India. In China, the genus *Belamcanda* comprises only one species, *B. chinensis*, the rhizome of which is utilized for medicinal purposes [1]. Dried whole herbs of *B. chinensis* are employed in traditional medicine in Guangdong, Guangxi, and several other areas in China [2]. The medicinal history of *B. chinensis* dates back to the Han Dynasty in China, where it was documented as a treatment for laryngeal paralysis and sore throat [2]. Historical records of traditional Chinese medicine indicate that *B. chinensis* is primarily used for its effects in clearing heat and detoxifying, as well as in alleviating phlegm and improving throat conditions [3]. Currently, more than ten proprietary Chinese medicines already on the market containing *B. chinensis* are included in the Chinese Pharmacopoeia (2020 Edition), with clinical applications addressing cough with phlegm, sore throat, chest tightness and abdominal distension. Another two approved Chinese patent medicines, She Gan Li Yan Kou Fu Ye and She Gan Kang Bing Du Zhu She Ye, utilize *B. chinensis* as a primary ingredient, specifically for antiviral purposes and sore throat treatment. Moreover, “She Gan Li Yan Kou Fu Ye” is derived from the “Shegansa” formula documented in the *Taiping Shenghui Fang* of the Song Dynasty, which is a national protected variety of traditional Chinese medicine, specifically designed to treat symptoms associated with children’s cough, as well as red and itchy throat, indicative and of lung and stomach heat [4].

In recent years, *B. chinensis* has been found to possess a structurally rich chemical composition as well as a variety of biological activities. To date, more than 200 compounds have been isolated from *B. chinensis*, including triterpenoids, flavonoids, phenolics, quinones, sesquiterpenoids, and steroids. Iridal-type triterpenoids, which are characteristic components of *B. chinensis* primarily consist of single-ring and spiral-ring structures. These compounds exhibit excellent anti-tumor, anti-inflammatory, hepatoprotective and kidney protective activity, and they also demonstrate potent ichthyotoxicity against killifish [5]. Isoflavones are the main structural types in *B. chinensis,* and also exert a variety of biological activities. Tectorigenin, a representative isoflavone component in *B. chinensis* that has been shown to regulate prostate-related gene products and inhibit tumor growth [6], scavenges DPPH free radicals [7] and inhibits liver damage caused by carbon tetrachloride to exert antioxidant and hepatoprotective activity [8]. It also exerts an increase in the cell viability of PC12 cells damaged by MPP+ to show neuroprotective activity [9]. In addition, macromolecular polysaccharides derived from natural plants have become a research hotspot in recent years because of their safety and low toxicity. There are eight polysaccharides isolated from dried rhizomes of *B. chinensis* that have performed structural characterization, and have been confirmed to indicate a potential application in the inhibition of liver cancer and anti-complementary activity [10,11,12,13].

Modern pharmacological activity studies have demonstrated that *B. chinensis* exhibits significant anti-inflammatory activity, which in turn has a potential therapeutic effect on diseases caused by inflammation [5]. Furthermore, *B. chinensis* has shown inhibitory effects on various tumor cells, offering additional therapeutic strategies for cancer treatment [14]. Additionally, it exhibits antioxidant [7], anti-diabetic [15] and hepatorenal protective activities [16,17]. There are also lesser-studied activities, including anti-VSMC proliferation [18] and anti-tussive, expectorant, analgesic [19] and anti-psoriatic effects [20], indicating that *B. chinensis* has the potential for drug development targeting these conditions. Moreover, toxicity studies have revealed that iridal-type triterpenoids in *B. chinensis* show some toxicity to *Oryzias latipes* and *Pomacea canaliculate*, while the extract demonstrates toxicity to *Brine Shrimp nauplii*.

Clinical studies have demonstrated that *B. chinensis* is predominantly utilized in conjunction with other medicinal herbs, either as part of a prescription or in combination with Western medicine, to treat various respiratory diseases, including bronchial asthma [21,22], cough variant asthma [23], chronic bronchitis [24] and chronic obstructive pulmonary disease [25]. Additionally, it has been employed in the treatment of other respiratory diseases conditions.

Therefore, we review the chemical composition, pharmacological activities, and toxicity of *B. chinensis*, to gain a comprehensive and systematic understanding of this plant and to provide a theoretical foundation for its future applications.

## 2. Materials and Methods

This review is the result of a comprehensive bibliographic analysis and aims to collate all available morphological, ethnomedical, phytochemical, pharmacological and toxicological studies on *B. chinensis*. Our literature search utilized a variety of scientific databases, including Sci-Finder, ScienceDirect, PubMed, China National Knowledge Infrastructure, Wiley, Springer Baidu Scholar and Sci-Hub, as well as domestic materia medica. The primary keyword used in this study is *Belamcanda chinensis*, paired with a relevant research area (e.g., chemical composition, pharmacology). The compiled bibliography included more than 100 references, ranging from original articles and reviews to essays, books, and book chapters, as well as websites covering publications from 1957 to 2025. We excluded unpublished papers and conference communications. All references included in this review were in Chinese and English and we accessed them in their entirety or through information abstracts. Chemical structures were cross-referenced and illustrated using ChemBioDraw Ultra 22.0 software.

## 3. Plant Description and Distribution

**Figure 1:**
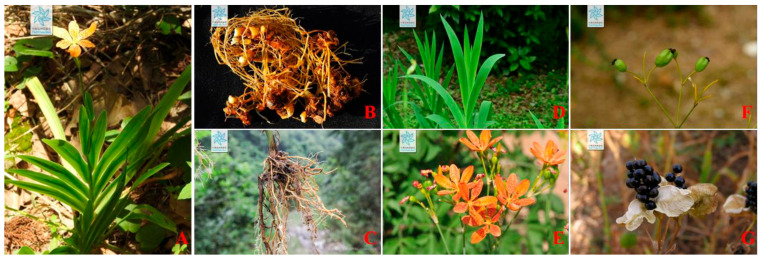
*B. chinensis*: (**A**) whole plant; (**B**) rhizomes; (**C**) roots; (**D**) flowers; (**E**) leaves; (**F**) fruits; (**G**) seeds. (The image is sourced from the iplant).

**Figure 2:**
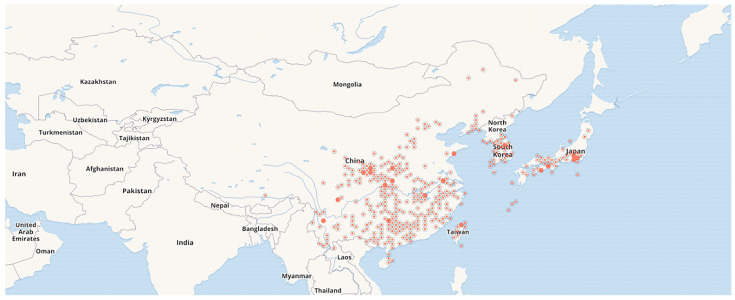
Map of the distribution of *B. chinensis* (GBIF, https://www.gbif.org/species/2748796, accessed on 29 November 2025).

*B. chinensis* is a perennial herb that reaches a height of 1 to 1.5 m. Its rhizomes are yellow to yellowish-brown, obliquely extended, and irregularly lumpy, with numerous yellowish fibrous roots. The leaves are sword-shaped, lack a midrib, and are arranged alternately in 2 rows, measuring 20 to 40 cm in length and 2 to 4 cm in width. The apical inflorescence exhibits forked branches, with membranous bracts at the junctions of the peduncles. The flowers are orange-red with purplish-brown spots. The perianth lobes are obovate or oblong-elliptic, with the inner whorls being slightly shorter and narrower than the outer whorls. The stamens have anthers that dehisce linearly outward and measure 1.8 to 2 cm in length, while the stigma is adorned with fine short hairs. The capsule is obovate, featuring an erect fruit shaft at its center, measuring 2.5 to 3 cm in length, and exhibits dorsal dehiscence along with outward-curving petals. The seeds are spherical, blackish-purple and glossy. The whole plant and morphological characteristics of its various parts of *B*. *chinensis* are presented in Figure 1. *B. chinensis* is widely distributed in China, primarily inhabiting low altitude areas such as forest edges or hillside grasslands, but it also thrives in the southwestern mountainous regions at altitudes of 2000 to 2200 m. Additionally, it is found in countries such as North Korea and Japan [1]. The global distribution of *B. chinensis* is shown in Figure 2.

## 4. Traditional Use

The rhizomes of the *B. chinensis* were used as an important medicine for the treatment of laryngeal paralysis in ancient times, the use of the rhizomes of *B. chinensis* as a medicine was recorded at the first time in the *Shen Nong Ben cao Jing* [26], where it was used to treat throat paralysis and sore throat and had the effect of clearing heat, relieving pain and relieving cough. It was recorded in *Hua Tuo Shen Fang* that *B. chinensis* can be used to treat menstrual irregularities and amenorrhea in women. It has been documented to dissipate knots, relieve pain and desilting in *Ming Yi Bie Lu* [27] and *Zhou Hou Bei Ji Fang* [28]. *Qian Jin Yi Fang* [29] has reported that it has an effect on eyesight. *Shang Han Zong Bing Lun* [30] and *Sheng Ji Zong Lu* [31] documented it has an expectorant effect. Additionally, it has been reported that it has been used to treat hot carbuncles. Among these works, the most representative is *Ben Cao Gang Mu* [32], where it is mentioned that *B. chinensis* is clinically used for the treatment of laryngeal paralysis and sore throat. Beyond the treatment of chest and flank fullness, women’s menstrual irregularities, and heat carbuncles, this work was also the first to propose that one mash the juice of the rhizomes of the *B. chinensis* and dispense it in that manner, in order to treat difficulties in urination and defecation. In addition, as a Thai medicinal plant, *B. chinensis* is used traditionally for the regulation of menstrual disorders [33]. Consequently, *B. chinensis* are used in pediatrics, gynecology, respiratory issues, carbuncles gangrene, sores, poison, scabies and other aspects.

According to the statistics of *Zhong Yi Fang Ji Da Ci Dian*, there are about 382 ancient formulas containing *B. chinensis*, most of which are used to treat heat-based diseases, and only 36 ancient formulas are used to treat cold-style diseases [34]. A total of representative prescriptions containing *B. chinensis* from various classics are summarized in Table 1.

Belamcandae Rhizoma is the dry rhizome of *B. chinensis*. When the stems and leaves first sprout in early spring or wither in late autumn, they are dug to remove fibrous roots and sediment and dried for subsequent use. It has the effects of clearing heat, detoxifying and eliminating phlegm, and relieving the pharynx (The Committee for the Pharmacopoeia of PR China, 2020) [35].

**Table 1:** The traditional prescriptions containing *B. chinensis*.

Preparation Name	Traditional Uses	References
Shegan Mahuang Tang	Cough with dyspnea and a wheezing sound in the throat	[36]
Shegan Tang	Cough in Cold Damage (Shanghan) syndrome, with throat obstruction and dysphagia	[37]
Shegan San (1)	The patient presented with polydipsia due to intense heat syndrome, accompanied by dorsal carbuncles, feverish dysphoria, and arthralgia	[38]
Shegan San (2)	The patient presented with toxic edema characterized by migratory lesions of uncertain origin.	[38]
Shegan Jian	The patient exhibited symptoms of wind-heat obstruction in the throat, including throat pain, dryness, and tongue rigidity, consistent with acute pharyngitis of wind-heat etiology	[38]
Shegan Shunianzi Tang	Cutaneous rash eruption with concomitant pharyngolaryngitis in pediatric patients	[39]
Shegan Wan	Beriberi heart disease complicating kidney yang deficiency syndrome	[37]

## 5. Phytochemical Compositions

**Figure 3:**
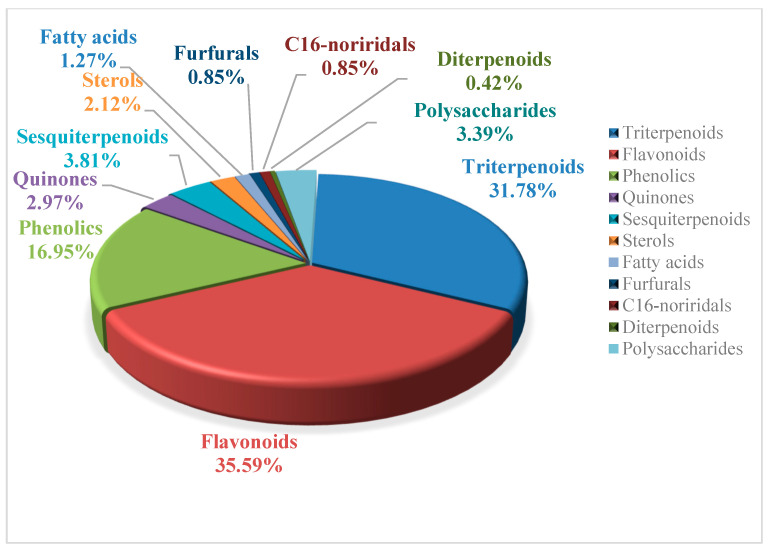
Proportion of different types of compounds in *B. chinensis* (%).

*B. chinensis* is rich in chemical components, which have been subjected to various separation and identification technologies to characterize their complex compositions. Currently, 228 chemical components have been isolated and identified from this plant, including triterpenoids, flavonoids, phenolics, quinones, sesquiterpenoids, and steroids, as well as eight polysaccharides. The proportion of different types of compounds in *B. chinensis* are shown in Figure 3. Among all of the components, triterpenoids and flavonoids are the primary constituents, predominantly found in the rhizomes of *B. chinensis.*

### 5.1. Triterpenoids

**Figure 4:**
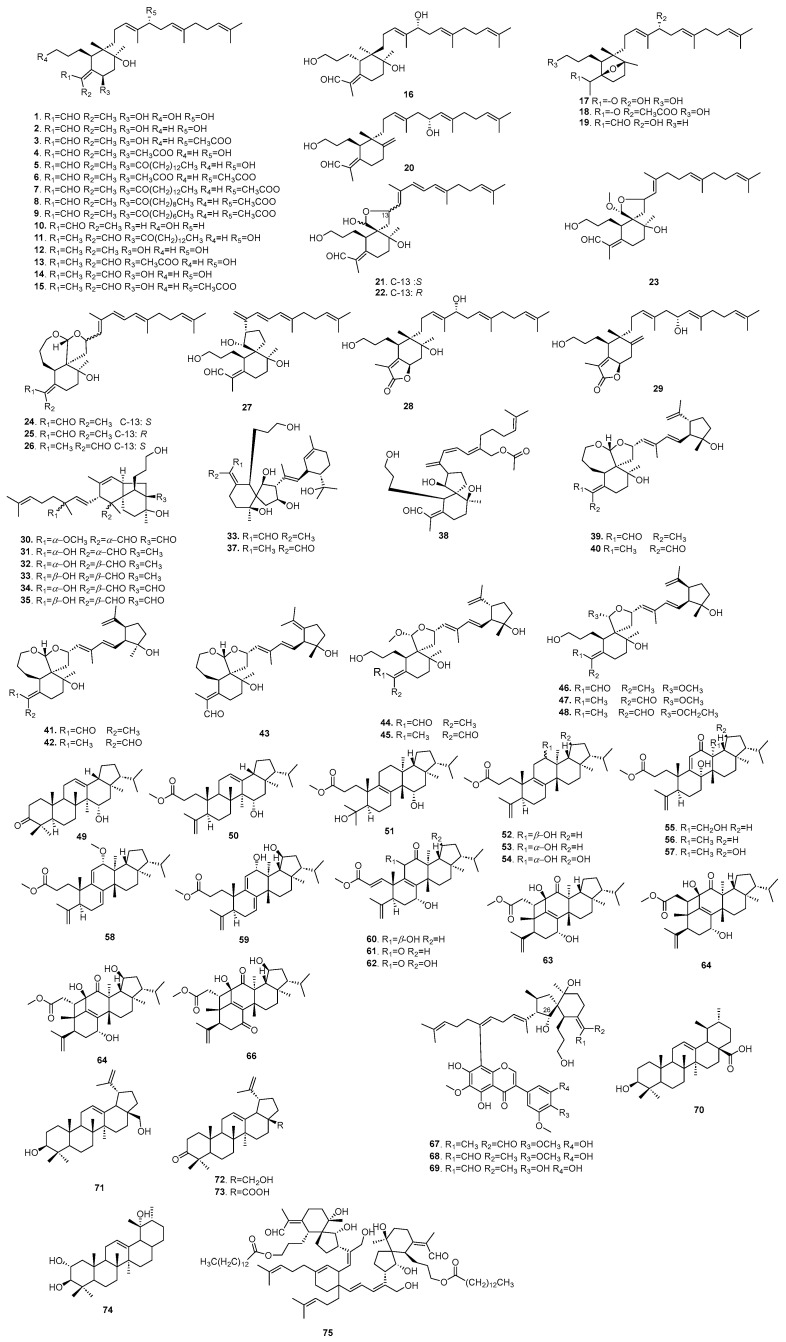
Chemical structures of triterpenoids (**1**–**75**) isolated from *B. chinensis*.

Triterpenoids are characterized by a fundamental structure consisting of 30 carbon atoms, derived from 6 isoprene units. To date, a total of 75 triterpenoids have been isolated from *B. chinensis*, including 55 iridal-type triterpenoids (**1**–**48**, **67**–**69**, **75**), which are recognized as the hallmark constituents of the Iridaceae family, primarily sourced from rhizomes and seeds. Additionally, there are 18 four-ringed triterpenoids (**49**–**66**) and 5 five-ringed triterpenoids (**70**–**74**). Inside the eight newly identified iridal-type triterpenoids (**1**, **4**, **12**, **16**–**18**, **28**, **29**) isolated from the rhizomes of *B. chinensis*, belamcanoxide B (**16**) has demonstrated moderate cytotoxicity against HCT-116 and MCF-7 cell lines [40]. Ichthyotoxic activity-guided fractionation of fresh rhizomes of *B. chinensis* has led to the isolation of six new iridal-type triterpenoids (**7**, **8**, **20**, **24**–**27**) alongside known triterpenoids (**5**, **22**). Among these compounds, 16-*O*-acetylisoiridogermanal A (**3**), belachinal (**21**), and spiroiridal (**22**) exhibited potent ichthyotoxic activity against killifish. Furthermore, the new dimeric iridal-type triterpenoid (**75**) from *B. chinensis* has been shown to possess significant molluscicidal activity. The components of triterpenoids are shown in Table 2, and their structures are shown in Figure 4.

**Table 2:** Isolation and identification of triterpenoids from *B. chinensis*.

NO.	Compound Name	Molecular Formula	Plant Parts	Extracts	References
**1**	8-Hydroxylisoiridogermanal	C_30_H_49_O_5_	Rhizomes	CH_2_Cl_2_	[40]
**2**	Iridobelamal A	C_30_H_50_O_4_	Rhizomes	CH_2_Cl_2_	[40]
**3**	16-*O*-Acetyliridobelamal A	C_32_H_52_O_5_	Rhizomes	CH_2_Cl_2_	[40]
**4**	3-*O*-Acetyliridobelamal A	C_32_H_52_O_5_	Rhizomes	CH_2_Cl_2_	[40]
**5**	Iristectorene B	C_44_H_76_O_5_	Rhizomes	Et_2_O	[40]
**6**	3,16-Di-*O*-acetylisoiridogermanal	C_34_H_54_O_6_	Rhizomes	Et_2_O	[5]
**7**	3-*O*-Tetradecanoyl-16-*O*-acetylisoIridogermanal	C_46_H_78_O_6_	Rhizomes	*n*-hexane	[5]
**8**	3-*O*-Decanoyl-16-*O*-acetylisoiridGermanal	C_42_H_70_O_6_	Rhizomes	*n*-hexane	[5]
**9**	3-*O*-Capryloyl-16-*O*-acetylisoiridogermanal	C_40_H_66_O_6_	Rhizomes	EtOAc	[16]
**10**	Iridal	C_30_H_50_O_4_	Twigs and leaves	EtOAc	[41]
**11**	Iristectorene A	C_44_H_76_O_5_	Rhizomes	EtOAc	[16]
**12**	Belamcandane A	C_30_H_52_O_3_	Rhizomes	CH_2_Cl_2_	[40]
**13**	3-*O*-Acetyliridobelamal B	C_32_H_52_O_5_	Rhizomes	CH_2_Cl_2_	[40]
**14**	Isoiridogermanal	C_30_H_50_O_4_	Rhizomes	CH_2_Cl_2_	[40]
**15**	16-*O*-Acetyl-iso-iridogermanal	C_32_H_52_O_5_	Rhizomes	CH_2_Cl_2_	[40]
**16**	Isoiridogermanal B	C_30_H_50_O_4_	Rhizomes	EtOH	[42]
**17**	Belamcanoxide B	C_29_H_48_O_4_	Rhizomes	CH_2_Cl_2_	[40]
**18**	16-*O*-Acetylbelamcanoxide B	C_31_H_48_O_5_	Rhizomes	CH_2_Cl_2_	[40]
**19**	Belamcanoxide A	C_30_H_50_O_4_	Twigs and leaves	EtOAc	[41]
**20**	Belamcandal A	C_30_H_48_O_3_	Rhizomes	CH_2_Cl_2_	[40]
**21**	Belachinal	C_30_H_46_O_5_	Rhizomes	Et_2_O	[5]
**22**	(6*R*,10*S*,11*R*)-26*ζ*-Hydroxy-(13*R*)-oxaspiroirid-16-enal	C_30_H_46_O_5_	Rhizomes	Et_2_O	[5]
**23**	Iridobelamal B	C_31_H_48_O_5_	Roots	*n*-hexane	[43]
**24**	Anhydrobelachinal	C_30_H_44_O_4_	Rhizomes	Et_2_O	[5]
**25**	Epianhydrobelachinal	C_30_H_44_O_4_	Rhizomes	Et_2_O	[5]
**26**	Isoanhydrobelachinal	C_30_H_44_O_4_	Rhizomes	Et_2_O	[5]
**27**	(+)-(6*R*,10*S*,11*S*,14*S*,26*R*)-26-Hydroxy-15-methylidenespiroirid-16-enal	C_30_H_46_O_4_	Rhizomes	Et_2_O	[5]
**28**	Belamcanolide B	C_30_H_48_O_5_	Rhizomes	CH_2_Cl_2_	[40]
**29**	Belamcanolide C	C_30_H_46_O_4_	Rhizomes	CH_2_Cl_2_	[40]
**30**	Belamchinenin A	C_31_H_48_O_5_	Rhizomes	EtOAc	[44]
**31**	Belamchinenin B	C_30_H_48_O_4_	Rhizomes	CH_2_Cl_2_	[45]
**32**	Belamchinenin C	C_30_H_46_O_5_	Rhizomes	CH_2_Cl_2_	[45]
**33**	Belamchinenin D	C_30_H_48_O_4_	Rhizomes	CH_2_Cl_2_	[45]
**34**	Belamchinenin E	C_30_H_46_O_5_	Rhizomes	CH_2_Cl_2_	[45]
**35**	Belamchinenin F	C_30_H_46_O_5_	Rhizomes	CH_2_Cl_2_	[45]
**36**	Belamcandane A	C_30_H_48_O_6_	Twigs and leaves	EtOAc	[46]
**37**	Belamcandane B	C_30_H_48_O_6_	Twigs and leaves	CH_2_Cl_2_	[47]
**38**	(6*R*, 10*S*, 11*S*, 14*S*, 26*R*)-(+)-29-Acetoxy-14, 15-dihydro-26-hydroxyspiroirida-15(28), 16-diena	C_44_H_74_O_5_	Rhizomes	EtOAc	[16]
**39**	Polycycloiridal K	C_30_H_44_O_5_	Rhizomes	CH_2_Cl_2_	[47]
**40**	Polycycloiridal L	C_30_H_44_O_5_	Rhizomes	CH_2_Cl_2_	[47]
**41**	Polycycloiridal M	C_30_H_44_O_5_	Rhizomes	CH_2_Cl_2_	[47]
**42**	Polycycloiridal N	C_30_H_44_O_5_	Rhizomes	CH_2_Cl_2_	[47]
**43**	Polycycloiridal O	C_30_H_44_O_5_	Rhizomes	CH_2_Cl_2_	[47]
**44**	Polycycloiridal P	C_31_H_48_O_6_	Rhizomes	CH_2_Cl_2_	[47]
**45**	Polycycloiridal Q	C_31_H_48_O_6_	Rhizomes	CH_2_Cl_2_	[47]
**46**	Polycycloiridal R	C_31_H_48_O_6_	Rhizomes	CH_2_Cl_2_	[47]
**47**	Polycycloiridal S	C_31_H_48_O_6_	Rhizomes	CH_2_Cl_2_	[47]
**48**	Polycycloiridal T	C_32_H_52_O_5_	Rhizomes	CH_2_Cl_2_	[47]
**49**	Belamcandaoid A	C_30_H_48_O_2_	Seeds	EtOAc	[17]
**50**	Belamcandaoid B	C_31_H_50_O_3_	Seeds	EtOAc	[17]
**51**	Belamcandaoid C	C_31_H_52_O_3_	Seeds	EtOAc	[17]
**52**	Belamcandaoid D	C_31_H_50_O_3_	Seeds	EtOAc	[17]
**53**	Belamcandaoid E	C_31_H_50_O_3_	Seeds	EtOAc	[17]
**54**	Belamcandaoid F	C_31_H_50_O_4_	Seeds	EtOAc	[17]
**55**	Belamcandaoid G	C_32_H_50_O_3_	Seeds	EtOAc	[17]
**56**	Belamcandaoid H	C_31_H_48_O_4_	Seeds	EtOAc	[17]
**57**	Belamcandaoid I	C_31_H_48_O_5_	Seeds	EtOAc	[17]
**58**	Belamcandaoid J	C_31_H_48_O_4_	Seeds	EtOAc	[17]
**59**	Belamcandaoid K	C_31_H_48_O_5_	Seeds	EtOAc	[17]
**60**	Belamcandaoid L	C_31_H_46_O_5_	Seeds	EtOAc	[17]
**61**	Belamcandaoid M	C_31_H_44_O_5_	Seeds	EtOAc	[17]
**62**	Belamcandaoid N	C_31_H_44_O_6_	Seeds	EtOAc	[17]
**63**	Belamchinane A	C_31_H_46_O_5_	Seeds	EtOAc	[48]
**64**	Belamchinane B	C_31_H_46_O_5_	Seeds	EtOAc	[48]
**65**	Belamchinane C	C_31_H_46_O_6_	Seeds	EtOAc	[48]
**66**	Belamchinane D	C_31_H_44_O_6_	Seeds	EtOAc	[48]
**67**	Belamcandanin A	C_48_H_62_O_13_	Twigs and leaves	EtOAc	[41]
**68**	Belamcandanin B	C_48_H_62_O_13_	Twigs and leaves	EtOAc	[41]
**69**	Belamcandanin C	C_47_H_60_O_12_	Twigs and leaves	EtOAc	[41]
**70**	Ursolic acid	C_30_H_48_O_3_	Roots	EtOAc	[49]
**71**	Betulin	C_30_H_50_O_2_	Roots	EtOAc	[49]
**72**	Betulone	C_30_H_48_O_2_	Roots	EtOAc	[49]
**73**	Betulonic acid	C_30_H_46_O_3_	Roots	EtOAc	[49]
**74**	2*α*,3*α*,19*α*-Trihydroxy-28-norurs-12-ene	C_29_H_48_O_3_	Rhizomes	EtOAc	[16]
**75**	Dibelamcandal A	C_88_H_144_O_12_	Rhizomes	EtOAc	[50]

### 5.2. Flavonoids

**Figure 5:**
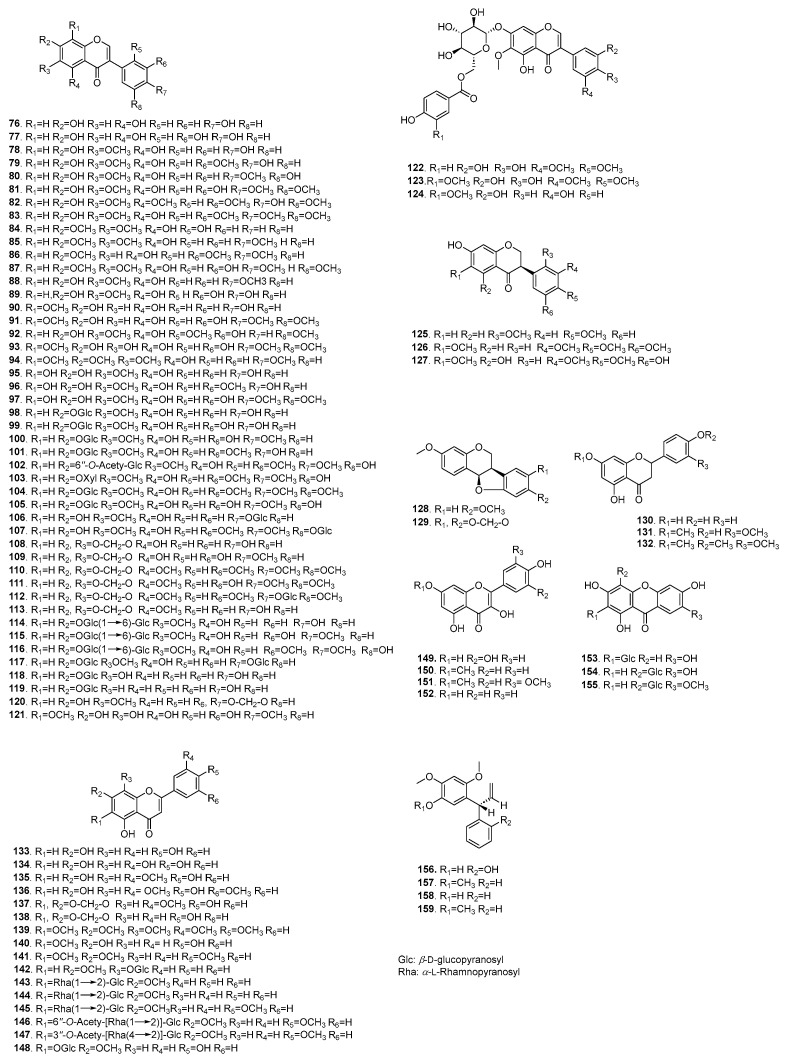
Chemical structures of flavonoids (**76**–**152**, **156**–**159**) and xanthones (**153**–**155**) isolated from *B. chinensis*.

Flavonoids are phenolic compounds derived from benzo-*γ*-pyrone, characterized by diverse structures and a wide distribution in the natural world. They exhibit a broad spectrum of pharmacological activities, including anti-inflammatory [51], antioxidant [52], hypoglycemic [53], estrogen receptor modulator [54] and anticancer [6] effects. To date, a total of 77 flavonoids have been isolated from various organs of *B. chinensis*. Among these, isoflavonoids represent the main structural type, comprising (**76**–**129**), followed by 23 flavones (**130**–**152**), 3 xanthones (**153**–**155**) and 4 neoflavonoids (**158**–**161**). There are 16 flavonoids isolated from the CH_2_Cl_2_ fraction of the rhizomes (**78**–**81**, **110**, **118**, **121**, **125**–**126**, **128**–**129**, **139**, **156**–**159**), of which tectorigenin (**78**), iristectorigenin B (**79**), irigenin (**81**), irisflorentin (**110**) and 5-*O*-demethylnobiletin (**139**) exert their inhibitory effects on the nitric oxide production in lipopolysaccharide (LPS)-induced Mouse leukemia cells of monocyte macrophage (RAW264.7) cells [51]. Additionally, four isoflavones (**108**, **114**–**115**, **124**) have been identified using on-line HPLC–DAD coupled with chemiluminescence and ESI-Q-TOF-MS/MS [52]. Notably, tectorigenin (**78**) possesses both estrogen receptor modulator and anticancer effects [6,54]. The components of flavonoids are shown in Table 3, and their structures are shown in Figure 5.

**Table 3:** Isolation and identification of flavonoids from *B. chinensis*.

NO.	Compound Name	Molecular Formula	Plant Parts	Extracts	References
**76**	Genistein	C_15_H_10_O_5_	Seeds	EtOAc	[55]
**77**	3′-Hydroxygenistein	C_15_H_10_O_6_	Seeds	EtOAc	[55]
**78**	Tectorigenin	C_16_H_12_O_6_	Rhizomes	CH_2_Cl_2_	[51]
**79**	Iristectorigenin B	C_17_H_14_O_7_	Rhizomes	CH_2_Cl_2_	[51]
**80**	Iristectorigenin A	C_17_H_12_O_7_	Rhizomes	CH_2_Cl_2_	[56]
**81**	Irigenin	C_18_H_16_O_8_	Rhizomes	CH_2_Cl_2_	[51]
**82**	5,7,4′-Trihydroxy-6,3′,5′-trimethoxyisoflavone	C_18_H_16_O_8_	Rhizomes	EtOAc	[57]
**83**	Junipegenin C	C_19_H_18_O_8_	Rhizomes	EtOAc	[57]
**84**	Irilin A	C_17_H_15_O_6_	Roots	EtOAc	[49]
**85**	4′,7-Dimethyltectorigenin	C_18_H_15_O_6_	Rhizomes	EtOAc	[16]
**86**	5-Hydroxy-7,3′,4′-trimethoxyisoflavone	C_18_H_15_O_6_	Rhizomes	EtOAc	[16]
**87**	Irigenin S	C_19_H_15_O_8_	Rhizomes	EtOAc	[16]
**88**	6-Hydroxybiochanin A	C_16_H_11_O_6_	Rhizomes	EtOAc	[16]
**89**	Irilin D	C_16_H_12_O_7_	Rhizomes	EtOAc	[58]
**90**	Isotectorigenin	C_16_H_12_O_6_	Rhizomas	CH_3_OH	[59]
**91**	Isoirigenin	C_16_H_12_O_6_	Rhizomes	EtOAc	[57]
**92**	5,7,3′-Trihydroxy-6,2′,5′-trimethoxyisoflavone	C_18_H_16_O_8_	Rhizomes	EtOAc	[14]
**93**	5,6,7,3′-Terahydroxy-8,4′,5′-trimethoxyisoflavone	C_18_H_16_O_9_	Rhizomes	EtOAc	[60]
**94**	6,7,8,4′-Tetramethoxy-5-hydroxyisoflavone	C_19_H_18_O_7_	Roots	EtOH	[61]
**95**	8-Hydroxytectrigenin	C_16_H_12_O_7_	Rhizomas	CH_3_OH	[59]
**96**	8-Hydroxyiristectrigenin A	C_17_H_14_O_8_	Rhizomas	CH_3_OH	[59]
**97**	8-Hydroxyirigenin	C_18_H_16_O_9_	Rhizomas	CH_3_OH	[59]
**98**	Tectoridin	C_22_H_22_O_11_	Leaves	CH_3_OH	[53]
**99**	3′-Hydorxytectoridin	C_22_H_22_O_12_	Rhizomes	EtOAc	[58]
**100**	Iristectorin A	C_23_H_24_O_12_	Rhizomes	EtOAc	[58]
**101**	Iristectorin B	C_23_H_25_O_12_	Rhizomes	CH_3_OH	[52,62]
**102**	Irigein-7-*O*-(6″-*O*-acetyl)-*β*-D-glucopyranoside	C_26_H_28_O_14_	Rhizomes	EtOAc	[63]
**103**	Irigenin-7-*O*-*β*-D-xylopyranoside	C_23_H_24_O_12_	Rhizomes	EtOAc	[63]
**104**	Iridin S	C_27_H_26_O_13_	Rhizomes	EtOAc	[14,63]
**105**	Iridin	C_26_H_24_O_13_	Leaves	*n*-hexane	[52,64]
**106**	Tectorigenin-4′-*O*-*β*-glucoside	C_22_H_22_O_11_	Rhizomes	EtOAc	[57]
**107**	Irigenin 3-*O*-*β*-glucopyranoside	C_24_H_25_O_13_	Aerial parts	EtOAc	[18]
**108**	Irilone	C_16_H_11_O_6_	Rhizomes	EtOAc	[60]
**109**	Iriflogenin	C_17_H_12_O_7_	Rhizomes	CH_3_OH	[52]
**110**	Irisflorentin	C_20_H_19_O_8_	Rhizomes	CH_2_Cl_2_	[51]
**111**	Dichotomitin	C_18_H_14_O_8_	Rhizomes	EtOH	[65]
**112**	3,5-Dimethoxy irisolone-4-*O*-*β*-D-glucoside	C_25_H_26_O_13_	Rhizomes	CHCl_3_	[66]
**113**	Nigricin A	C_17_H_12_O_6_	Rhizomes	EtOAc	[63]
**114**	Tectorigenin-7-*O*-*β*-glucosyl (1⟶6) glucoside	C_28_H_32_O_16_	Rhizomes	CH_3_OH	[52]
**115**	Iristectorigenin B-7-*O*-*β*-glucosyl (1⟶6) glucoside	C_29_H_34_O_17_	Rhizomes	CH_3_OH	[52]
**116**	Irigein-7-[*O*-*β*-D-glucopyranosyl-(1⟶6)-*β*-D-glucopyranoside]	C_22_H_23_O_11_	Rhizomes	CH_3_OH	[67]
**117**	Tectorigenin-7-*O*-glucosyl-4′-*O*-glucoside	C_28_H_32_O_16_	Rhizomes	CH_3_OH	[52]
**118**	Dalspinin	C_17_H_12_O_7_	Rhizomes	CH_2_Cl_2_	[56]
**119**	Genistin	C_21_H_20_O_10_	Leaves	CH_3_OH	[53]
**120**	Daidzin	C_21_H_20_O_9_	Leaves	CH_3_OH	[53]
**121**	3′,5,7-Trihydroxy-8,4′ dimethoxyisoflavone	C_17_H_14_O_8_	Rhizomes	CH_2_Cl_2_	[56]
**122**	6″-*O*-*p*-Hydroxybenzoyliridin	C_31_H_30_O_15_	Rhizomes	Et_2_O	[68]
**123**	6′-*O*-Vanilloyliridin	C_32_H_32_O_16_	Rhizomes	Et_2_O	[68]
**124**	Tectorigenin-7-*O*-[6″-*O*-(3‴-methoxy-4‴-hydroxyl-benzoyl]-*β*-D-glucopyranoside	C_29_H_25_O_13_	Rhizomes	CHCl_3_	[64]
**125**	Sativanone	C_17_H_16_O_5_	Rhizomes	CH_2_Cl_2_	[51]
**126**	3′-*O*-Methylviolanone	C_18_H_18_O_6_	Rhizomes	CH_2_Cl_2_	[51]
**127**	2,3-Dihydroirigenin	C_18_H_18_O_8_	Seeds	EtOAc	[55]
**128**	Pterocarpin	C_17_H_14_O_5_	Rhizomes	CH_2_Cl_2_	[51]
**129**	Homopterocarpin	C_17_H_16_O_4_	Rhizomes	CH_2_Cl_2_	[51]
**130**	5,7,4′-Trihydroxyflavnone	C_15_H_12_O_5_	Rhizomes	EtOH	[69]
**131**	5,4′-Dihydroxy-7,3′-dimethoxyflavanone	C_17_H_15_O_6_	Rhizomes	EtOAc	[16]
**132**	5-Hydroxy-7,3′,4′-trimethoxyflavanone	C_18_H_17_O_5_	Rhizomes	EtOAc	[16]
**133**	Apigenin	C_15_H_10_O_5_	Rhizomes	EtOH	[65,70]
**134**	Luteolin	C_15_H_10_O_6_	Rhizomes	EtOH	[70]
**135**	Isorhamnetin	C_16_H_12_O_7_	Rhizomes	CHCl_3_	[51]
**136**	5,7,4′-Trihydroxy-3′,5′-dimethoxyflavone	C_17_H_14_O_7_	Rhizomes	EtOH	[70]
**137**	5,4′-Dihydroxy-6,7-methylenedioxy-3′ -methoxyflavone	C_17_H_12_O_7_	Rhizomes	CHCl_3_	[66]
**138**	Kanzakiflavone-2	C_16_H_10_O_6_	Rhizomes	EtOAc	[57]
**139**	5-*O*-Demethylnobiletin	C_20_H_20_O_8_	Rhizomes	CH_2_Cl_2_	[51]
**140**	Hispiludin	C_16_H_12_O_6_	Rhizomes	CH_3_OH	[59]
**141**	Dimethyltectorigenin	C_18_H_16_O_6_	Rhizomes	EtOAc	[16]
**142**	Isoswertisin	C_22_H_22_O_10_	Aerial parts	EtOAc	[18]
**143**	2″-*O*-*α*-L-Rhamnosyl-4′-*O*-methylisovitexin	C_28_H_33_O_15_	Aerial parts	EtOAc	[18]
**144**	2″-*O*-Rhamnosylswertisin	C_28_H_32_O_14_	Aerial parts	EtOAc	[18]
**145**	Embinin	C_22_H_22_O_10_	Aerial parts	EtOAc	[18]
**146**	6″-*O*-Acetylembinin	C_31_H_35_O_15_	Aerial parts	EtOAc	[18]
**147**	3″-*O*-Acetylembinin	C_31_H_35_O_15_	Aerial parts	EtOAc	[18]
**148**	Swertisin	C_22_H_22_O_10_	Leaves	CH_3_OH	[53]
**149**	Quercetin	C_15_H_10_O_7_	Seeds	EtOAc	[55]
**150**	Rhamnocitrin	C_16_H_12_O_6_	Rhizomes	Et_2_O	[68]
**151**	Rhamnazin	C_17_H_14_O_7_	Rhizomes	CHCl_3_	[66]
**152**	Kampferol	C_15_H_10_O_6_	Roots	EtOAc	[49]
**153**	Mangiferin	C_19_H_18_O_11_	Leaves	CH_3_OH	[53]
**155**	Isomangiferin	C_19_H_18_O_11_	Rhizomes	CH_3_OH	[71]
**154**	7-*O*-Methylisomangiferin	C_20_H_20_O_11_	Rhizomes	CH_3_OH	[71]
**156**	Latifolin	C_17_H_18_O_4_	Rhizomes	CH_2_Cl_2_	[51]
**157**	5-*O*-Methyllatifolin	C_18_H_20_O_4_	Rhizomes	CH_2_Cl_2_	[51]
**158**	Dalbergiphenol	C_17_H_18_O_3_	Rhizomes	CH_2_Cl_2_	[51]
**159**	5-*O*-Methyldalbergiphenol	C_18_H_20_O_3_	Rhizomes	CH_2_Cl_2_	[51]

### 5.3. Phenolics

**Figure 6:**
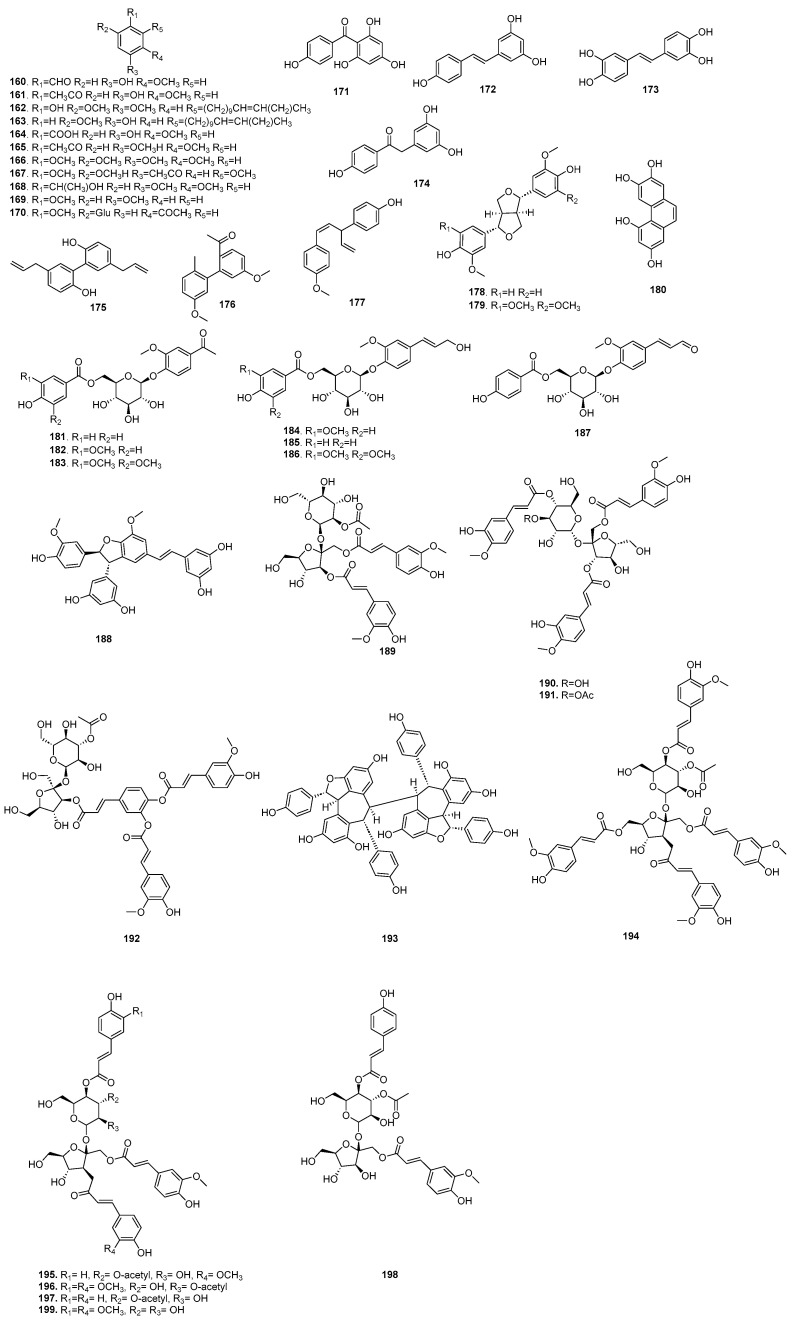
Chemical structures of phenolics (**160**–**199**) isolated from *B. chinensis*.

Phenolics represent a significant class of aromatic compounds, which are mainly formed by the substitution of hydrogen by hydroxyl groups on the aromatic ring, and their structural types are rich and diverse. Up to now, 38 phenolics (**160**–**188**) and their derivatives have been mainly obtained from the CH_2_Cl_2_ and EtOAc extracts of the different parts of *B. chinensis*. There are 11 phenolics (**160**–**170**), which contain only 1 benzene that has been isolated from the rhizomes and seeds. Furthermore, vanillin (**160**) and apocynin (**161**) exhibited significant inhibitory effects of NO production in RAW 264.7 cells with IC_50_ values in the range of 18.0–52.6 µM. The 10 sucrosephenylpropanoid esters (**184**–**187**, **189**–**194**) have been isolated from the whole plants of *B. chinensis.* The components of phenolics are shown in Table 4, and their structures are shown in Figure 6.

**Table 4:** Isolation and identification of phenolics from *B. chinensis*.

NO.	Compound Name	Molecular Formula	Plant Parts	Extracts	References
**160**	Vanillin	C_8_H_8_O_3_	Rhizomes	CH_2_Cl_2_	[51]
**161**	Apocynin	C_9_H_10_O_3_	Rhizomes	CH_2_Cl_2_	[51]
**162**	Belamcandol A	C_23_H_38_O_3_	Seeds	CH_2_Cl_2_	[72]
**163**	Belamcandol B	C_22_H_36_O_2_	Seeds	CH_2_Cl_2_	[72]
**164**	4-Hydroxyl-3-methoxyl benzoic acid	C_8_H_8_O_4_	Rhizomes	EtOH	[65]
**165**	(3, 4-Dime-thoxyphenyl)-ethanone	C_10_H_12_O_3_	Rhizomes	EtOAc	[16]
**166**	1, 2, 4, 5-Tetramethoxybenzene	C_10_H_14_O_4_	Rhizomes	EtOAc	[16]
**167**	3,4,5-Trimethoxyacetophenone	C_11_H_14_O_4_	Rhizomes	EtOAc	[16]
**168**	1-(3, 4-Dimethoxyphenyl) ethanol	C_10_H_13_O_3_	Rhizomes	EtOAc	[16]
**169**	1, 4-Dimethoxybenzene	C_8_H_10_O_2_	Rhizomes	EtOAc	[16]
**170**	Androsin	C_15_H_20_O_8_	Rhizomes	EtOAc	[73]
**171**	Iriflophenone	C_13_H_10_O_5_	Rhizomes	EtOAc	[58]
**172**	Resveratrol	C_12_H_14_O_3_	Rhizomes	EtOAc	[58]
**173**	Piceatannol	C_14_H_12_O4	Rhizomes	EtOH	[62]
**174**	Belamphenone	C_14_H_12_O_4_	Rhizomes	EtOAc	[58]
**175**	Magnolol B	C_18_H_18_O_2_	Rhizomes	EtOAc	[63]
**176**	2′-Acetyl-4′,4-dimethoxybiphenyl-2-carbaldehyde	C_17_H_16_O_4_	Rhizomes	EtOAc	[16]
**177**	4′-*O*-Methylnyasol	C_18_H_18_O_2_	Rhizomes	EtOAc	[52]
**178**	Pinoresinol	C_20_H_22_O_6_	Rhizomes	EtOH	[14]
**179**	(+)-Syringaresinol	C_22_H_26_O_8_	Seeds	EtOAc	[55]
**180**	Phenanthrenetriol A	C_14_H_10_O_4_	Rhizomes	EtOAc	[63]
**181**	Belalloside B	C_22_H_24_O_16_	Rhizomes	EtOAc	[58]
**182**	Belalloside A	C_23_H_26_O_11_	Rhizomes	EtOAc	[58]
**183**	Apocynin-4-*O*-*β*-D-(6′-*O*-syringyl)glucopyranoside	C_23_H_31_O_12_	Rhizomes	EtOAc	[63]
**184**	Diplostephioside C	C_26_H_32_O_13_	Rhizomes	EtOAc	[63]
**185**	Diplostephioside B	C_25_H_30_O_12_	Rhizomes	EtOAc	[63]
**186**	Diplostephioside D	C_23_H_26_O_10_	Roots	EtOAc	[69]
**187**	Diplostephioside E	C_23_H_24_O_10_	Roots	EtOAc	[69]
**188**	Shegansu B	C_30_H_26_O_8_	Roots	EtOAc	[74]
**189**	2′-*O*-Acetyl-1,3-*O*-diferuloylsucrose	C_33_H_32_O_18_	Aerial parts	EtOAc	[18]
**190**	Belamcanoside A	C_42_H_46_O_20_	Seeds	EtOAc	[55]
**191**	Belamcanoside B	C_44_H_48_O_21_	Seeds	EtOAc	[55]
**192**	Shegansu C	C_44_H_48_O_21_	Rhizomes	CHCl_3_	[75]
**193**	(−)-Hopeaphenol	C_56_H_42_O_12_	Seeds	EtOAc	[55]
**194**	Belamchinoside A	C_30_H_26_O_8_	Roots	CH_2_Cl_2_	[76]
**195**	Belamchinoside B	C_30_H_26_O_8_	Roots	CH_2_Cl_2_	[76]
**196**	Belamchinoside C	C_30_H_26_O_8_	Roots	CH_2_Cl_2_	[76]
**197**	Belamchinoside D	C_30_H_26_O_8_	Roots	CH_2_Cl_2_	[76]
**198**	Belamchinoside E	C_30_H_26_O_8_	Roots	CH_2_Cl_2_	[76]
**199**	Belamchinoside F	C_30_H_26_O_8_	Roots	CH_2_Cl_2_	[76]

### 5.4. Miscellaneous Compounds

**Figure 7:**
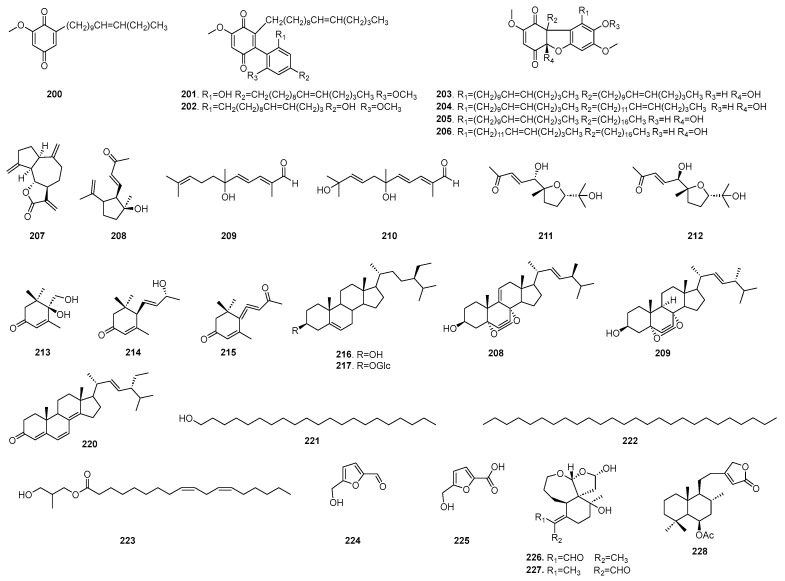
Chemical structures of miscellaneous compounds (**200**–**228**) isolated from *B. chinensis*.

In addition to the above compounds, quinones, sesquiterpenoids, diterpenoids, sterols, furfuraloids and C_16_-noriridals have also been isolated from *B. chinensis*. Until now, the seven quinones (**200**–**206**), which are 1,4-benzoquinones, have all been derived from the seeds of *B. chinensis*. The nine sesquiterpenoids (**207**–**215**) and one diterpenoid (**228**) have been isolated from the rhizomes and roots. The five sterols (**216**–**220**) are obtained from the EtOAc extracts of rhizomes and roots. Additionally, 5-hydroxymethyl-2-furaldehyde (**224**) and 5-hydroxymethyl-2-formyl-pyrrol (**225**) have been isolated from the rhizomes of *B. chinensis*, which contain a furan ring, an aldehyde group and a hydroxymethyl group in the molecule. This is an important fine chemical raw material for the preparation of various derivatives through oxidation, hydrogenation and condensation reactions. In addition, two C_16_-noriridals (**226**–**227**) have been isolated from the rhizomes of *B. chinensis*. The components of miscellaneous compounds are shown in Table 5, and their structures are shown in Figure 7.

**Table 5:** Isolation and identification of miscellaneous compounds from *B. chinensis*.

NO.	Compound Name	Molecular Formula	Plant Parts	Extracts	References
**200**	Belamcandaquinone	C_22_H_34_O_3_	Seeds	*n*-hexane	[77]
**201**	Aquinone A	C_44_H_68_O_5_	Seeds	EtOH	[78]
**202**	Aquinone B	C_44_H_68_O_5_	Seeds	EtOH	[78]
**203**	Belamcandone A	C_44_H_68_O_7_	Seeds	*n*-hexane	[77]
**204**	Belamcandone B	C_46_H_72_O_7_	Seeds	*n*-hexane	[77]
**205**	Belamcandone C	C_46_H_74_O_7_	Seeds	*n*-hexane	[77]
**206**	Belamcandone D	C_48_H_78_O_7_	Seeds	*n*-hexane	[77]
**207**	Dehydrocostus lactone	C_15_H_18_O_2_	Rhizomes	CH_2_Cl_2_	[51]
**208**	Belchinoid A	C_13_H_22_O_2_	Roots	EtOH	[61]
**209**	Belchinoid B	C_14_H_22_O_2_	Roots	EtOH	[61]
**210**	Belchinoid C	C_13_H_22_O_3_	Roots	EtOH	[61]
**211**	Belchinoid D	C_13_H_23_O_3_	Rhizomes	EtOAc	[16]
**212**	Belchinoid E	C_13_H_23_O_3_	Rhizomes	EtOAc	[16]
**213**	Crocusatin M	C_10_H_23_O_3_	Rhizomes	EtOAc	[16]
**214**	(6*R*,7*E*,9*R*)-9-Hydroxy-4,7-megastigmadien-3-one	C_13_H_19_O_2_	Rhizomes	EtOAc	[16]
**215**	3*S*,5*R*-Dihydroxy-6*S*,7-megastigmadien-9-one	C_13_H_16_O_2_	Rhizomes	EtOAc	[16]
**216**	*β*-Sitosterol	C_29_H_50_O	Roots	EtOAc	[49]
**217**	Daucosterol	C_35_H_60_O_3_	Roots	EtOAc	[49]
**218**	(22*E*,24*S*)-5*α*,8*α*-Epidioxy-24-methyl-cholesta-6,9(11), 22-trien-3*β*-ol	C_28_H_42_O_3_	Rhizomes	EtOAc	[16]
**219**	(22*E*)-5*α*, 8*α*-Epidioxyergosta-6, 22-dien-3*β*-ol	C_28_H_44_O_3_	Rhizomes	EtOAc	[16]
**220**	Stigmata-4,6,8(14),22-tetraen-3-one	C_29_H_42_O	Rhizomes	EtOAc	[16]
**221**	Heneicosanol	C_21_H_44_O_1_	Rhizomes	EtOAc	[16]
**222**	Glycerin linoleate	C_26_H_54_	Rhizomes	EtOAc	[16]
**223**	Hexacosane	C_22_H_44_O_3_	Rhizomes	EtOAc	[16]
**223**	5-Hydroxymethyl- 2-furaldehyde	C_6_H_6_O_3_	Rhizomes	EtOH	[65]
**225**	5-(Hydroxymethyl)-2-formyl-pyrrol	C_6_H_6_O_4_	Rhizomes	EtOAc	[16]
**226**	Irispseudoacorin A	C_16_H_27_O_5_	Rhizomes	EtOAc	[16]
**227**	Irispseudoacorin B	C_16_H_27_O_5_	Rhizomes	EtOAc	[16]
**228**	Vitexilactone	C_22_H_34_O_5_	Rhizomes	CH_2_Cl_2_	[51]

### 5.5. Polysaccharides

Polysaccharides are natural, large-molecule, long-chain polymers composed of more than ten monosaccharide units linked by glycosidic bonds, exhibiting a variety of biological activities [79]. Currently, eight polysaccharides have been isolated from the dried rhizomes of *B. chinensis*. These polysaccharides demonstrate varying degrees of anti-liver cancer activity, complement inhibition, and offer significant cytoprotective effects on PC12 cells [10,11,12,13]. The components of these polysaccharides are detailed in Table 6.

## 6. Pharmacological Activity

### 6.1. Anti-Inflammatory Activity

**Figure 8:**
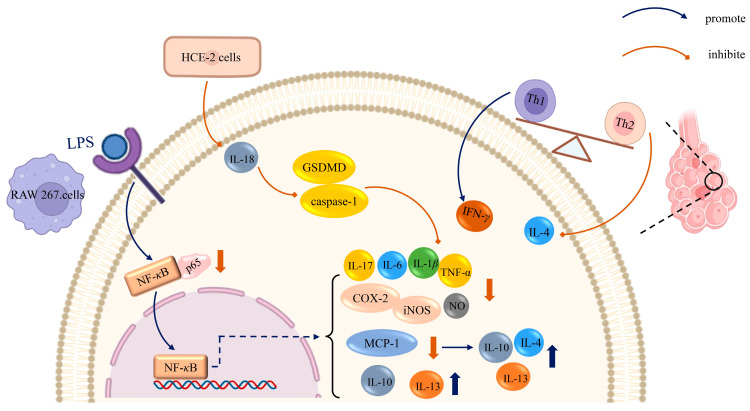
Molecular mechanisms of *B. chinensis* on anti-inflammatory activities.

Inflammation is the body’s response to cellular damage or infection, and serves as a precursor to various diseases, including cancer, diabetes, cardiovascular disease, and depression. All phases of the inflammatory reaction are response by external cellular mediators, including cytokines, chemokines, growth factors, eicosanoids (such as prostaglandins), and complement proteins. Recent in vivo and in vitro studies have demonstrated that extracts and monomeric compounds derived from *B. chinensis* exhibit anti-inflammatory activity through diverse pathways, thereby expanding the therapeutic strategies available for treating inflammation-induced diseases [19]. The molecular mechanisms of B. chinensis on anti-inflammatory activities are shown in Figure 8.

Mouse leukemia cells of monocyte macrophage (RAW264.7) were derived from tumors induced by Abelson’s leukemia virus (A-MuLV) in male BALB/c mice. Under the action of lipopolysaccharide (LPS), RAW264.7 cells will mimic the inflammatory response and release or up-regulate a variety of inflammatory mediators, such as nitric oxide (NO), cyclooxygenase-2 (COX-2), tumor necrosis factor (TNF), interleukin-6 (IL-6). This serves as the most commonly used in vitro research model for screening anti-inflammatory actives. To date, many compounds isolated from *B. chinensis* have been evaluated for their anti-inflammatory effects against RAW264.7 cells in vitro. Jeong et al. isolated isoiridogermanal, iridobelamal A and iridobelamal B from the roots of *B. chinensis* and assessed their inhibitory activities against human neutrophil elastase (HNE) using a spectrophotometric method. Their results indicated that all three compounds exhibited selective inhibition of HNE. Then, they used RNA isolation and reverse transcriptase (RT)-polymerase chain reaction (PCR) to test the expression of proinflammatory cytokines, their results showed that isoiridogermanal and iridobelamal A displayed significant anti-inflammatory effects by suppressing the expressions of inducible nitric oxide synthase (iNOS), interleukin-1β (IL-1β), and TNF in LPS-stimulated RAW 264.7 cells. Then Western Blot analysis with nuclear and cytoplasmic fractions were used to judge nuclear translocation of the p65 protein, which further determined that the isolates exert anti-inflammatory effects by suppressing the expressions proinflammatory cytokines through the nuclear factor-κB (NF-κB) pathway in LPS-stimulated RAW 264.7 cells. This study represented the first report identifying iridal-type triterpenoids as the responsible phytochemicals for anti-inflammatory effects of *B. chinensis* [43]. Nine isolated compounds from the rhizomes of *B. chinensis* have been shown to inhibit NO production in RAW 264.7 macrophage cells, with IC_50_ values ranging from 8.8 to 52.6 μM. Here, the sesquiterpene lactone demonstrated the most potent inhibitory effect, with an IC_50_ value of 8.8 μM, which is better than the positive drug, aminoguanidine, which had an IC_50_ value of 17.5 μM [51]. Seok et al. demonstrated irigenin dose-dependently suppressed LPS-induced NO and prostaglandin E2 production. Moreover, this compound reduced the expression of inducible iNOS and COX-2 proteins and mRNAs without significant cytotoxicity [80]. Festus identified a 50% or greater inhibition rate against COX-2 in LPS-stimulated RAW264.7 cells for 13 compounds isolated from the CH_2_Cl_2_ extract of *B. chinensis*. Here, 3′,5,7-trihydroxy-8,4′-dimethoxyisoflavone showed the strongest inhibition rate, with an IC_50_ value of 4.20 µM. Additionally, iristectorigenin A, iristectorigenin B and dalspinin demonstrated moderate activities with IC_50_ values of 7.23 µM, 7.84 µM, and 8.20 µM, respectively [56]. Qian evaluated the activity of 23 monomeric compounds isolated and identified from the roots of *B. chinensis* in vitro. It was found that 6 compounds, including 4 iridal-type triterpenoids, 1 phenolic and 1 steroid, exhibited a significant inhibitory effect on the LPS-induced release of NO from RAW 264.7 macrophages, with the inhibitory activity of 15-dihydro-26-hydroxyspiroirida-15(28)-16-diena showing IC_50_ values of 0.56 ± 0.04 µM, which is substantially higher than that of the positive control drug, PDTC (with IC_50_ values of 4.83 ± 0.13 µM). Additionally, among these compounds, two of them, iridal-type triterpenoid and steroid, demonstrated an inhibitory effect on IL-6 [16]. To investigate anti-inflammatory activity and mechanism in vitro, LPS-induced RAW264.7 macrophages were employed as the inflammatory model. Guo et al. discovered that the isoflavones iristectorin A and iristectorin B modulated the cholinergic anti-inflammatory pathway, the arachidonic acid metabolic pathway and the NO pathway. These compounds can reduce the release of inflammatory mediators such as NO, TNF, IL-1β and IL-6 in macrophages, thereby achieving a multi-pathway anti-inflammatory effect [81].

Liu et al. employed a luciferase assay to determine the inhibition rates against the NF-κB signaling pathway, thereby assessing the anti-inflammatory activities of *B. chinensis*. At the concentration of 10 μM, the phenolic compounds, including tectoridin A, nigricin A and naringenin, from the rhizomes of *B. chinensis* demonstrated inhibition rates of 53.71%, 57.68% and 88.71% against the NF-κB signaling pathway, respectively, in SW480 human colon cancer cells [63]. In the no cytotoxicity range, concentrations of 10, 20 or 40 µM, irigenin protected the human corneal epithelial cell line against hyperosmolarity-induced inflammation and dysfunction by reducing interleukin-18 (IL-18) levels, enhancing cell viability, proliferation and migration, and inhibiting the apoptosis in hyperosmolarity-induced human comeal epithelial cells-2 (HCE-2 cells). Consequently, irigenin showed potential as a candidate for the development of therapeutic agents for dry eye disease [82].

Furthermore, mangiferin was found to reduce both the macro- and microscopic damage scores and the malondialdehyde (MDA) levels in colon tissues. At a dosage of 100 mg/kg, mangiferin significantly decreased the concentrations of TNF and IL-17, as well as superoxide dismutase (SOD) activity in colon tissues. Therefore, mangiferin is able to relieve 2,4,6-trinitro-benzenesulfonicacid (TNBS)-induced colitis in rats [83].

The polar extraction method was employed to separate the *B. chinensis* extract into chemical component groups based on their differing polarities. Subsequently, pharmacodynamics tests were conducted to assess anti-inflammatory effects on these chemical component groups. The results indicated that a high dose of *B. chinensis* extract significantly inhibited the swelling rate of rat paws at the peak period of one hour post-injection of the egg white, showing a notable difference when compared with the model control group (*p* < 0.05), which suggests its anti-inflammatory properties. Moreover, both the ethyl acetate extract and *n*-butanol extract exhibited a pronounced antagonistic effect during the early stage of the swelling peak [19]. Furthermore, RSV infection in guinea pigs resulted in a thickening of the alveolar walls, a significant increase in inflammatory cells, and elevated levels of interleukin-4 (IL-4), while the level of interferon-γ (IFN-γ) was markedly reduced. The intervention with *B. chinensis* extract significantly decreased the total number of inflammatory cells in bronchoalveolar lavage fluid (BALF), increased the level of IFN-γ, and decreased the levels of IL-4 and leukotriene C4 (LTC4) in the bronchoalveolar lavage fluid (BALF) supernatant. These findings support the potential application of *B. chinensis* in alleviating lung inflammation [84]. Yang et al. investigated the anti-inflammatory activities of the ethanol extract of *B. chinensis* (EEBC) and its underlying mechanisms. The results demonstrated that intraperitoneal injection of EEBC at doses of 100 and 200 mg/kg significantly reduced carrageenan-induced foot swelling and xylene-induced ear swelling in mice. Additionally, at doses of 100 and 200 mg/kg, EEBC markedly decreased LPS-induced NO secretion, inhibited iNOS activity, and lowered the levels of TNF, IL⁃1β, and IL-6 [85]. Feng investigated the potential mechanisms by which *B. chinensis* extract may ameliorate polycystic ovary syndrome (PCOS) through inflammatory pathways. Following BCE treatment, a notable decrease in the total macrophage population and M1 infiltration was observed, which corresponded with reduced levels of IL-6, IL-12, IL-1β, and NO. Additionally, an increase in M2 infiltration was noted, while the expression of chemokine-1 in ovarian tissues decreased. These findings suggest that the improvement of ovarian polycystic morphology in polycystic ovary syndrome (PCOS) mice following BCE treatment may be associated with the reduction of M1 infiltration, decreased expression of monocyte chemoattractant protein-1 (MCP-1), and increased infiltration of M2 macrophages in the ovaries [86].

The aforementioned studies indicate that extracts and compounds derived from *B. chinensis* greatly inhibit the release or up-regulation variety of inflammatory mediators such as NO, COX-2, TNF, IL-6 and IL-1β caused by LPS-induced microglial activation, moreover, some compounds have better inhibitory activity than positive drugs. Additionally, the potential of the regulation of the NF-κB signaling pathway to exert the Anti-inflammatory activity has been proven. Furthermore, *B. chinensis* demonstrated promising therapeutic effects on conditions such as psoriasis, lung inflammation, dry eye syndrome, and polycystic ovary syndrome caused by inflammation. The molecular mechanisms of B. chinensis on anti-inflammatory activities are shown in Figure 8.

### 6.2. Anti-Tumor Activity

**Figure 9:**
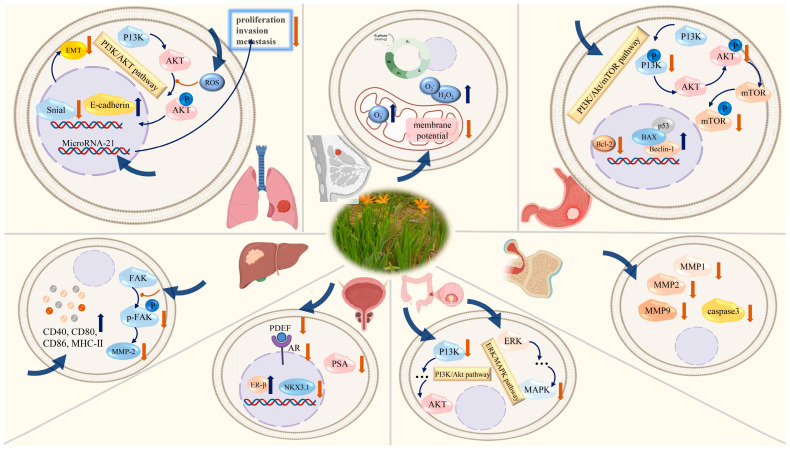
Molecular mechanisms of *B. chinensis* on anti-tumor activities.

Cancer represents a significant global health challenge and continues to lack completely effective treatments. Compounds derived from *B. chinensis* have been shown to inhibit several cancer cell lines by inhibiting proliferation and migration and by inducing apoptosis, including those associated with breast, liver, colon, stomach, and prostate cancers. The molecular mechanisms of *B. chinensis* on anti-tumor activities are shown in Figure 9. Three iridal-type triterpenoids, isolated from the rhizomes of *B. chinensis*, exhibited moderate cytotoxic activities against three human cancer cell lines: HCT-116, HepG2, and MCF-7. Specifically, belamcanoxide B demonstrated cytotoxic activities against the HCT-116 and MCF-7 cell lines, with IC_50_ values of 5.58 and 3.35 μM, respectively, while 16-*O*-acetylisoiridogermanal displayed moderate cytotoxic activities against the HepG2 and MCF-7 cell lines, with IC_50_ values of 7.66 and 6.43 μM, respectively. Furthermore, 3-*O*-acetyliridobelamal A exhibited moderate cytotoxicity against the HCT-116 and HepG2 cell lines, with IC_50_ values of 8.71 and 7.22 μM, respectively. These findings suggest that the iridal-type triterpenoids containing an ether bridge moiety may play a critical role in their cytotoxic activities [40]. Ni et al. discovered a novel tricyclic-fused triterpenoid from *B. chinensis* named belamchinenin A, which features a flexible geranyl side chain attached to a rigid half-caged nucleus scaffold system. The cytotoxic activities of belamchinenin A were evaluated against NCI-H1650, HepG2, BGC-823, HCT-116, and MCF-7 cells using the MTT assay, demonstrating significant cytotoxic effects against several cancer cell lines [44]. Similarly, four iridal-type triterpenoids, namely belamcanoxide A, iridobelamal A, isoiridogermanal, and iridal, exhibited moderate cytotoxic activities against five cancer cell lines, with IC_50_ values ranging from 3.26 to 8.63 μM [41]. Liu et al. reported that pentacyclic triterpenes derived from the ethyl acetate extract demonstrated moderate inhibitory activities, exhibiting a synergistic effect against the growth of human carcinoma cell lines. Notably, ursolic acid inhibited the growth of MGC-803 cells by inducing apoptosis in tumor cells [49]. These findings provide further evidence that iridals possess significant cytotoxic activities against cancer cell lines and represent promising candidates for the development of new anticancer drugs.

Triple-negative breast cancer (TNBC) is a highly malignant tumor characterized by strong invasiveness, a high degree of deterioration, and poor prognosis, accounting for 15–20% of all breast cancers. All TNBC cases are negative for progesterone receptor (PR), estrogen receptor (ER), and human epidermal growth factor receptor 2 (Her-2). Currently, chemotherapy is the primary treatment for TNBC. Exploring active components in natural plant resources presents a feasible strategy for TNBC management. Commonly used triple-negative breast cancer cell lines include, but are not limited to, MDA-MB-231 and MDA-MB-468. Recent findings indicate that extracts from *B. chinensis* possess the potential to combat triple-negative breast cancer. Ten compounds were isolated from the rhizomes of *B. chinensis*, including three phenolic acids, three isoflavones, two iridal-type triterpenoids, one flavonoid and one sterol, all of which exhibited significant antiproliferation effects across five breast cancer cell lines (BT549, 4T1, MCF7, MDA-MB-231, and MDA-MB-468). Notably, isoiridogermanal demonstrated the highest activity against the 4T1 and MDA-MB-468 cell lines. Further flow cytometry was used in studies to reveal that isoiridogermanal acts by inhibiting cell metastasis, inducing cell cycle arrest in the G1 phase, down-regulating mitochondrial membrane potential, and generating excessive reactive oxygen species (ROS) to induce apoptosis in 4T1 and MDA-MB-468 cells. In summary, these findings suggest that isoiridogermanal holds promising potential for the treatment of triple-negative breast cancer and warrants further evaluation [14]. Additionally, Yi employed the CCK8 method to trace the active components of *B. chinensis* against triple-negative breast cancer, revealing that both isoiridogermanal and isoiridogermanal B exhibited anti-proliferative effects on five breast cancer cell lines (MDA-MB-468, MDA-MB-23, MCF-7, BT549, 4T1) with the most pronounced inhibitory effect observed on MDA-MB-468 cells, with IC_50_ values of 15.74 ± 1.04 μM and 26.97 ± 2.68 μM. These compounds significantly inhibited the proliferation and migration of MDA-MB-468 cells in a dose-dependent manner and arrested the cell cycle of these cells in the G1 phase. Furthermore, they induced apoptosis in MDA-MB-468 cells by significantly decreasing mitochondrial membrane potential and increasing ROS levels [42].

The 4′-*O*-methylnyasol from the rhizomes of *B. chinensis*, classified as a dihydroflavonol, demonstrated an antiproliferative activity of 84.91% against the K562 human leukemia cell line, with an IC_50_ value of 4.20 μM [63].

Prostate cancer is the most prevalent malignancy among men, characterized by significant dietary influences and an extended latency period. Therefore, it is crucial to delay the initiation or progression of the disease. Thelen et al. used real time RT-CR, ELISA, TRAP assays and Western Blots to measure the antiproliferative effects of isoflavones on cancer cells. Notably, the isoflavone tectorigenin effectively corrects the aberrant expression of several essential gene products involved in prostate cancer, including pleiotropic transcription factor (PDEF), prostate specific antigen (PSA), insulin like growth factor-1(IGF-1) receptor mRNA, and hTERT mRNA, and in vitro studies have shown that telomerase activity is down-regulated, while TIMP-3 mRNA is up-regulated. Furthermore, the growth of subcutaneous tumors in nude mice administered extracts from *B. chinensis* was significantly delayed and diminished. These findings suggest that isoflavonoids such as tectorigenin etc. from *B. chinensis* represent valuable targets for future applications in prostate cancer management [6]. Additionally, Thelen et al. reported that *Belamcanda chinensis* extracts, as phytoestrogens, exhibit chemopreventive properties in prostate cancer by decreasing the expression of the androgen receptor (AR) and its coactivator PDEF, concomitant with reduced cell proliferation and PSA secretion, as well as the expression of NKX3.1 [87].

Chen et al. established a mouse model of inflammatory associated colorectal cancer by the chemical induction of azoxymethane (AOM) and dextran sulfate sodium (DSS), and used the ELISA method to investigate the effect of *B. chinensis* extract on the inflammatory response related to intestinal cancer in AOM/DSS mice as well as its potential its possible mechanism. The results demonstrate a significant reduction in the levels of signal transducer and activator of transcription 3 (STAT3) and IL-6 proteins in the serum of the mice. The mechanism of action appears to be linked to the inhibition of the IL-6/STAT3 signaling pathway. Furthermore, the study reveals that *B. chinensis* extract also decreases the expression levels of mitogen-activated protein kinases (MAPK) and the phosphoinositide 3-kinase (PI3K) in the serum of AOM/DSS model mice, thereby inhibiting the progression of enteritis to intestinal cancer by modulating the ERK/MAPK and PI3K/Akt signaling pathways [88,89].

The genesis and development of lung adenocarcinoma is a complex process involving multiple factors, steps, and genes. Tectoridin acts on lung adenocarcinoma cells, causing the production of reactive oxygen species (ROS), which in turn inhibits the PI3K/AKT pathway. This action leads to the down-regulation of Snail expression and the inhibition of epithelial-mesenchymal transition (EMT), ultimately resulting in reduced tumor cell migration [90]. Similarly, the ethanol extract of *B. chinensis* significantly inhibits both the growth and invasion capabilities of H460 lung cancer cells by markedly down-regulating the expression of microRNA-21 in these cells [91].

Liu et al. investigated the effects of tectoridin on autophagy and apoptosis in the human gastric cancer cell line HGC-27 by CCK-8, clone formation test, flow cytometry and Western blotting. Their results indicated that tectoridin inhibited the proliferation of HGC-27 cells in a time- and dose-dependent manner, promoted autophagy and apoptosis, up-regulated the protein expression of the autophagy factor Beclin-1, and the apoptosis factors p53 and BAX, while decreasing the protein expression of the anti-apoptotic molecule B-cell lymphoma-2 (Bcl-2). Furthermore, they used KEGG enrichment assay to determine that the inhibition of HGC-27 cell growth is associated with the suppression of the PI3K/Akt/mTOR signaling pathway, which induced apoptosis and autophagy [92]. Zhao et al. isolated an undisclosed polysaccharide, BCP80-2, and activity assays demonstrated that BCP80-2 significantly suppressed the growth, metastasis, and angiogenesis of HepG2 cells in zebrafish. Mechanistic studies have indicated that BCP80-2 inhibited the migration of HepG2 cells by suppressing the FAK signaling pathway. Additionally, BCP80-2 activated immunomodulation and up-regulated the secretion of co-stimulatory molecules CD40, CD86, CD80, and MHC-II [11]. Furthermore, Zhao et al. coupled BCP50-2 with gold nanorods to gain BCP50-2-AuNRs, which had a great photothermal conversion effect. Under near-infrared (NIR) light irradiation, this exhibited a great effect in suppressing the growth of HepG2, A549, and MCF-7 cells. Additionally, it was able to inhibit tumor proliferation, migration, and angiogenesis in zebrafish [10].

Guo et al. assessed the anti-tumor effects of tectorigenin against osteosarcoma and investigated its underlying biological mechanisms. They found that tectorigenin inhibited the proliferation of osteosarcoma cells (Saos2 and U2OS) in a dose- and time-dependent manner. Furthermore, tectorigenin significantly inhibited migration and invasion in osteosarcoma cells, up-regulating the expression of cleaved cysteinyl aspartate specific proteinase-3 (caspase-3) while down-regulating the expression of matrix metalloproteinases, including MMP1, MMP2, and MMP9 [93].

In vivo and in vitro studies have demonstrated that the active compounds of *B. chinensis* exhibit significant antitumor activity by inhibiting tumors through various mechanisms, including cell cycle arrest and the induction of apoptosis. These results provide further evidence that *B. chinensis* may serve as a promising candidate for the development of new anticancer drugs.

### 6.3. Antioxidant and Antimutagenic

Song evaluated the antioxidant activity of belamcanosides A and B in relation to cholesterol synthesis and metabolism at the gene level. The findings indicated that belamcanosides A and B could regulate the expression of genes associated with cholesterol synthesis and metabolism, including 3-hydroxy-3-methylglutaryl-coenzyme A reductase (HMGCR), squalene epoxidase (SQLE), low-density lipoprotein receptor (LDLR), and sortilin (SORT1), in HepG2 cells [55]. Additionally, the antioxidant activities of iridal-type triterpenoid derivatives, characterized by a 6/5/6 tricyclic ring skeleton and isolated from the rhizomes of *B. chinensis*, were assessed using an MDA colorimetric assay in Fe^2+^/cysteine-induced liver microsomal lipid peroxidation. The results demonstrated that belamchinenin C-F effectively suppressed Fe^2+^/cysteine-induced liver microsomal lipid peroxidation at a concentration of 10 μM [45].

Zhang et al. firstly evaluated the capacity to scavenge DPPH free radicals to investigate the antioxidant activity of compounds from *B. chinensis*, using vitamin C as a positive control. They found that dichotomintin, irigenin, tectorigenin, and iridin could scavenge DPPH free radicals with IC_50_ values of 22.8, 190.1, 76.6, and 28.5, and 10.2 mg/mL, respectively. Furthermore, the antioxidant activity of iridin was found to surpass that of vitamin C (VC) [7]. Additionally, Festus evaluated antioxidant activity using three different assays: DPPH, Ferric ion reducing antioxidant power (FRAP), and ABTS. The results indicated that the isoflavones dalspinin, iriatorigenin B and 3′,5,7-trihydroxy-8,4′-dimethoxyisoflavone exhibited the strongest DPPH radical scavenging activity (IC_50_ = 10.44 µM), highest ABTS free radical scavenging activity (IC_50_ = 13.49 µM), and strongest FRAP resistance (4.77 µM Fe^2+^), respectively, and where dalspinin outperformed the positive control, vitamin C [56].

With VC as a positive control, Lu evaluated the reducing power of total terpenes from the fruit, stem, and beard of *B. chinensis* on FeCl_3_, as well as the DPPH and ABTS^+^ free radical scavenging rates to assess their antioxidant capacity. The results indicated that all samples exhibited certain reducing abilities, with fruit total terpenes demonstrating the most significant activity in DPPH and ABTS^+^ free radical scavenging [94].

### 6.4. Neuroprotective Activity

Stroke poses a significant threat to human health and is recognized as one of the major diseases affecting the population. Consequently, there is increasing interest in natural anti-stroke drugs, which are noted for their efficacy and reduced side effects. Evaluating the activity of chemical components in these natural drugs is of the great significance. The PC12 cell activity screening technique was employed to preliminarily assess the neuroprotective effects of the crude extract of *B. chinensis*. The results indicated a significant enhancement in the cell viability of MPP^+^-injured PC12 cells. Subsequently, lactate dehydrogenase inhibitors were accurately screened from the crude extract of *B. chinensis* using ultrafiltration mass spectrometry. The study identified tectoridin, iridin, tectorigenin, irigenin, and iriflorentin as compounds that could biologically interact with lactate dehydrogenase, thereby inhibiting its biological activity. This finding suggests that isoflavones derived from *B. chinensis* possess potential anti-stroke activity [9]. Furthermore, Zhou analyzed and verified the effects of iristectorin B on the proteomics of an ischemic stroke cell model to explore its protective mechanisms on PC12 cells. The results demonstrated that iristectorin B significantly reduced PC12 cell injury induced by oxygen-glucose deprivation/reoxygenation (OGD/R), decreased apoptosis, increased cell survival rates, and lowered the levels of Ca^2+^, lactate dehydrogenase (LDH), and ROS. Proteomic analysis revealed that it could modulate differentially expressed proteins such as heme ooxygenase 1 (HMOX1), transferrin receptor protein 1 (TFR1), and solute carrier family 3 member 2 (SLC3A2), which are primarily involved in the regulation of ferroptosis [95]. Additionally, Duan et al. employed alcohol gradient ethanol precipitation combined with column chromatography separation technology to obtain four polysaccharides. In order to evaluate the protective effects of the PC12 cells of the four polysaccharides, they established an OGD/R model and tested the cell viability via the CCK-8 method. The result suggested that BCP50-1a, BCP70-1a, and BCP90-1a exhibited protective effects against the injury that OGD/R induces in PC12 cells [12]. Although no studies to date have investigated compounds or extracts derived from *B. chinensis* in the MACO model, other plant-derived compounds or extracts have demonstrated efficacy in ameliorating MCAO-induced acute cerebral ischemic injury [96,97], suggesting its potential value for further research in vivo.

Liu elucidated the underlying molecular mechanisms by which tectorigenin-treated neuron-like NT2/D1 cells significantly induced the expression of Erythropoietin (EPO) mRNA through the accumulation of hypoxia inducible factor-1α (HIF-1α) in both cultured neuron-like NT2/D1 cells and rat cortical neurons, thereby playing a crucial role in the treatment of these cultured neurons, whereby tectorigenin up-regulation of EPO in neurons works to exhibit the neuroprotective function [98]. Sylwester Ślusarczyk employed an ELISA microtiter assay to evaluate the inhibitory effects of various compounds on acetylcholinesterase (AChE) and butyrylcholinesterase (BchE). The results indicated that piceatannol exhibited inhibition of both cholinesterases by up to 67% and 91%, respectively, while irilin D and resveratrol were selectively effective against BchE. Additionally, Irisflorentin was identified as a weakly selective inhibitor of AchE. Consequently, these compounds were proposed as potential lead structures for cholinesterase inhibition and dementia management [62]. In an effort to develop new effective therapeutic interventions for alleviating Parkinson’s disease (PD)-associated pathologies, Guo et al. explored the effects of irigenin on MPP^+^-induced BV-2 cells. The results demonstrated that irigenin attenuated the MPP^+^-induced increase in malondialdehyde content and the activities of superoxide dismutase, catalase, and glutathione peroxidase in BV-2 cells, while also suppressing apoptosis, caspase-3/7 activity, and cytochrome C expression. Furthermore, irigenin activated the kelch-like ECH-associated protein 1(Keap1)/nuclear factor erythroid 2-related factor 2 (Nrf2) pathway in MPP^+^-induced BV-2 cells, which was crucial for alleviating MPP^+^-induced neurotoxicity [99].

### 6.5. Hypoglycemic Activity

In the study conducted by Wu et al., the *α*-glucosidase inhibitory effects of the aqueous leaf extract of *B. chinensis* (BCL) and its rough isoflavone preparation (BIF) were evaluated both in vitro and in vivo. Male Kunming mice were administered BCL at doses of 500 mg/kg and 1000 mg/kg, and BIF at doses of 250 mg/kg and 500 mg/kg, respectively. The results indicated that both BCL and BIF significantly inhibited the increase in blood glucose levels in normal mice one hour after starch intake, demonstrating a dose-dependent relationship. Subsequently, the α-glucosidase inhibitory activity was assessed in vitro, revealing that six isoflavones isolated from BCL exhibited strong α-glucosidase inhibitory activity, with swertisin identified as the principal active component, with an IC_50_ value of 119 µg/mL [53]. Yuan investigated the biological activity of *B. chinensis*, screening 24 compounds including 9 phenolics, 7 isoflavones and 4 flavones for *α*-glucosidase inhibitory activity, among which 5 compounds, including 19*α*-Trihydroxy-28-norurs-12-ene, 6-hydroxybiochanin A, 5,4′-dihydroxy-7,3′-dimethoxyflavanone, 5-hydroxy-7,3′,4′-trimethoxyflavanone and *trans*-resveratrol, inhibited *α*-glucosidase by more than 50% [16]. Li utilized ultrafiltration mass spectrometry to discover that tectorigenin, irigenin, iristectorigenin A, and irisflorentin could bind to the *α*-glucosidase receptor, thereby exhibiting inhibitory effects on α-glucosidase [9]. Yan isolated the aqueous extract of *B. chinensis* leaves and explored the spectrum-effect relationships between HPLC chromatograms and the hypoglycemic activities of various isolates from the leaf extract. The study concluded that flavonoids were the primary contributors to the hypoglycemic activity [100].

Aldose reductase (AR), a key enzyme in the polyol pathway, is essential for preventing diabetic complications. Jung evaluated the active principles for aldose reductase inhibition derived from the rhizomes of *B. chinensis* by measuring rat lens AR activity. The compounds tectoridin and tectorigenin exhibited the highest inhibitory potency, exhibiting IC_50_ values of 1.08 µM for both. These compounds were administered orally at a dosage of 100 mg/kg to streptozotocin-induced diabetic rats over a period of 10 consecutive days, resulting in a significant reduction of sorbitol accumulation in tissues such as the lens, sciatic nerves, and red blood cells [101].

Guo et al. investigated the effects of the extract of *B. chinensis* leaves on obesity-induced diabetes using KK-A^y^ mice. Their findings revealed that component F2, which was precipitated from 95% isopropanol of BCLE, alleviated hyperglycemia and insulin resistance, as evidenced by decreased levels of fasting blood glucose (FBG), area under the curve (AUC), glycosylated serum protein (GSP), lactate dehydrogenase (LDH), and insulin. Furthermore, F2 was shown to inhibit glycogen synthase kinase-3 beta (GSK-3β) and enhance liver glycogen levels, demonstrating that F2 could play a significant role in inhibiting hepatic gluconeogenesis and promoting glycogen accumulation. Additionally, an increase in peroxisome proliferator-activated receptor gamma (PPAR γ) was observed, indicating that F2 may prevent insulin resistance via the PI3K signaling pathway. Moreover, component F1 of BCLE was found to prevent cell degeneration and reduce pathological tissue injury in pancreatic tissue. Collectively, these results suggest that F2 exhibits substantial hypoglycemic activity and could be further explored as a potential therapeutic agent for type 2 diabetes mellitus [15]. Then, they investigated the lipid-lowering effects of BCLE on obese diabetic KK-A^y^ mice, revealing that component F2 markedly ameliorated lipid disorders and decreased body weight, liver index, and levels of triglyceride (TG), total cholesterol (TC), and low-density lipoprotein cholesterol (LDL-c) in serum and liver. Additionally, it exhibited significant antioxidant activity by enhancing liver SOD and inhibiting MDA levels. Moreover, the supernatant component F1 reduced TG, LDL-c, and MDA levels in the liver. These results suggest that F2 may have therapeutic potential in the prevention and treatment of hyperlipidemia and liver disease associated with obesity-related diabetes [15].

*B. chinensis* is a traditional Chinese herbal medicine, supported by these studies for its remarkable hypoglycemic activity, underscoring its potential in diabetes treatment.

### 6.6. Other Activities

**Figure 10:**
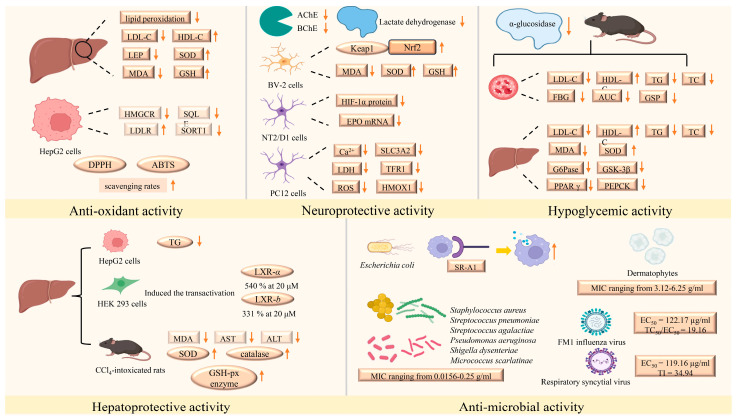
Molecular mechanisms of *B. chinensis* on other activities.

In addition to the aforementioned activities, studies have demonstrated that *B. chinensis* exhibits hepatoprotective, antibacterial, and antiviral estrogenic properties, as well as anti-psoriatic and nephroprotective activities etc. The molecular mechanisms of *B. chinensis* on other activities are shown Figure 10.

Jun et al. carried out transfection and luciferase assays to establish that iristectorigenin B functions as a novel liver X receptor (LXR) modulator, significantly inducing the transactivation of both LXR-α (540% at 20 μM) and LXR-β (331% at 20 μM) in a dose-dependent manner in HEK 293 cells. Furthermore, they used cholesterol efflux experiments and quantitative PCR to determine that iristectorigenin B enhances cholesterol efflux by inducing ATP binding cassette transporter A1(ABCA1) and ATP binding cassette subfamily G Member 1 (ABCG1), thereby demonstrating hypocholesterolemic effects without inducing hepatic steatosis [102]. Yuan developed a hyperlipidemic model, using HepG2 cells with 0.5 mM oleic acid for 24 h, to assess the inhibition of triglyceride accumulation by compounds derived from *B. chinensis*. The results indicated that two norsesquiterpenes (belchinoid A-B), one isoflavone (6,7,8,4′-tetramethoxy-5-hydroxy isoflavone), and three iridal triterpenoids (3-*O*-capryloyl-16-*O*-acetylisoiridogermanal, 16-*O*-acetyliridobelamal A, and anhydrobelachinal) exhibited significant lipid-lowering activity, suggesting their potential for the treatment of nonalcoholic fatty liver disease [16]. Li et al. utilized *B. chinensis* as a precursor to synthesize nanometer components of its charcoal via a high-temperature pyrolysis method, termed BRC-NCs. They subsequently established an acute liver injury model induced by carbon tetrachloride (CCl4) in mice to investigate the hepatoprotective effects of Belamcande Rhizoma Carbonisatum nano components (BRC-NCs). The results indicated that BRC-NCs significantly reduced serum levels of alanine aminotransferase (ALT), aspartate aminotransferase (AST), direct bilirubin (DBIL), and indirect bilirubin (IBIL), as well as MDA in liver homogenate, while markedly increasing SOD levels in liver homogenate (*p* < 0.05) and alleviating liver pathological damage. This study represents the first identification of BRC-NCs from Carbonisatum and confirms their significant role in acute liver injury. This finding not only expands the application range of nano-compounds but also provides a reference for the clinical treatment of liver injury [103]. To investigate the antioxidant activities of isoflavones derived from the rhizomes of *B. chinensis* on carbon tetrachloride-induced hepatic injury in rats, Jung et al. assessed the serum activities of AST and ALT, hepatic antioxidative enzyme activities, and levels of lipid peroxidation in tectorigenin and tectoridin. They found that these compounds significantly reduced the levels of MDA, AST, and ALT in CCl_4_-intoxicated rats, while also restoring the activities of superoxide dismutase SOD, catalase, and glutathione peroxidase GSH-px impaired by CCl_4_. These findings suggest that tectorigenin and tectoridin isolated from *B. chinensis* possess not only antioxidative properties but also hepatoprotective effects in CCl_4_-intoxicated rats [8].

Bacterial infections are a significant threat to patients in intensive care units (ICUs), serving as a primary contributor to life-threatening organ dysfunction and the progression to sepsis. Consequently, it is essential to investigate medicinal therapies targeting bacterial infections. Li et al. conducted a study examining the sensitivity of extracts from *B. chinensis* with varying polarities against various bacterial strains. Their findings indicated that *Streptococcus pneumoniae* and *Pseudomonas aeruginosa* exhibited sensitivity to the extracts of *B. chinensis*, with inhibition zones exceeding 19 mm in diameter. Furthermore, the extracts significantly decreased the mortality in mice caused by *Staphylococcus aureus* yeast suspension, demonstrating a bacteriostatic effect in vivo [19,104]. Xiang investigated the potential of irisflorentin (IFL) as a treatment for diseases caused by bacterial infections, such as sepsis. The study demonstrated that IFL could suppress the inflammatory response induced by methicillin-resistant staphylococcus aureus (MRSA) or a synthetic mimic of bacterial lipoprotein (Pam3CSK4). Additionally, IFL was found to up-regulate the expression of the phagocytic receptors SR-A1 and FcγR2a, thereby enhancing the phagocytic ability of macrophages against bacteria. Furthermore, IFL inhibited the secretion of IL-6 and TNF induced by heat-killed MRSA in mouse bone marrow-derived macrophages. Therefore, we speculate that IFL has the potential to be a leading compound for host-directed therapy in the treatment of bacterial infections [105]. Jahan et al. used the disc diffusion method to evaluate the antibacterial activity of *B. chinensis* extract. The results indicated that tectorigenin exhibited strong antifungal activity against dermatophytes of the genus Trichophyton, with a minimum inhibitory concentration (MIC) ranging from 3.12 to 6.25 mL [106].

*B. chinensis* exhibits properties that clear heat and eliminate toxins. Dong investigated the antiviral activity of the acetyl acetate extract fraction derived from the ethanol extract of the rhizomes of *B. chinensis*. Initially, the researcher evaluated its effect on the influenza virus in chick embryos. The results indicated that, compared with the control group, the virus titer in the experimental group decreased by more than 2 logs, demonstrating a significant inhibitory effect on the titers of both the influenza A virus and the influenza B virus. Subsequently, the antiviral activity was tested in mice. Under mild ether anesthesia, mice were infected with 20 LD_50_ of the virus via nasal drops. The findings revealed that the extract provided a protective effect against the death of mice caused by the influenza virus and extended the survival time of the mice [20]. Li et al. employed a cytopathic effect inhibition assay (CPEI) to evaluate the anti-influenza virus FM1 and anti-RSV effects of the *B. chinensis* extract. The results demonstrated that the *B. chinensis* extract exhibited a significant inhibitory effect on both FM1 and RSV, with the EC_50_ value of 122.17 µg/mL and 119.16 µg/mL [19].

Nowak et al. initially investigated the antiosteoporotic properties of mangiferin, focusing on bone turnover, bone mineral density (BMD), and bone microarchitecture in vivo. They measured a range of serological markers of the bone turnover and histomorphometric parameters of the proximal tibial metaphysis. Their findings indicate that mangiferin inhibits the reduction of tibial BMD and enhances bone microarchitecture in the ovariectomized group. Additionally, normalization of the beta *C*-terminated telopeptide of type I collagen (bCTX) level was observed in mangiferin-treated rats [107]. Meanwhile, Ga et al. investigated the mechanism by which irilin D (IRD) inhibits receptor activator of NF-κB ligand (RANKL)-induced osteoclast formation and function. Mechanistically, IRD disrupts the RANKL-induced activation of MAPKs and NF-κB, resulting in the inhibition of c-Fos and nuclear factor of activated T cells cytoplasmic 1 (NFATc1) activation. Furthermore, IRD suppresses the expression of RANKL-induced NFATc1 target genes, including dendrocyte expressed seven transmembrane protein (DC-STAMP), artrate resistant acid phosphatase 5 (ACP5), and cathepsin K (CtsK). Therefore, it is suggested that IRD mitigates LPS-induced inflammatory bone resorption in mice by inhibiting RANKL-activated MAPKs and NF-κB signaling pathways, indicating its potential as a natural isoflavone for the prevention or treatment of osteoclast-associated diseases [108]. Manh Tuan Ha conducted an initial investigation into the anti-osteoclastogenic activity of compounds derived from *B. chinensis*. Among all of the compounds studied, belamchinoside A and irilin D demonstrated the most significant inhibitory effects on the formation of TRAP-positive osteoclasts in RAW264.7 cells, effectively reducing the RANKL-induced expression of NFATc1 in a concentration-dependent manner [76].

In the search for novel estrogenic compound-derived plants, Orawan investigated the extract of the rhizomes of *B. chinensis*. Among the compounds identified, resveratrol, iriflophenone, tectorigenin, tectoridin, and belamphenone were found to stimulate the proliferation of both MCF-7 and T-47D human breast cancer cells [58]. D. Seidlova confirmed the selective estrogen receptor modulator activities of tectorigenin. The study demonstrated that tectorigenin induced transactivation in ERα-expressing MCF-7 cells and ERβ-expressing MDA-MB-231 reporter gene-transfected cells. Furthermore, it inhibited pulsatile pituitary LH secretion in ovariectomized (ovx) rats and was able to sustain uterine weight as well as estrogen-regulated uterine gene expression [54].

Song et al. evaluated the biological activities associated with the kidney protection offered by belamcandaoid C and belamcandaoid M, which were isolated from the seeds of *B. chinensis*. The results indicated that belamcandaoid C and belamcandaoid M could respectively reduce the expression of fibronectin and collagen I in TGF-β1-induced kidney proximal tubular cells [17]. Furthermore, they demonstrated that belamchinanes A–D, also isolated from the seeds of *B. chinensis*, provide dose-dependent protection against age-related renal fibrosis in vitro [48].

To investigate the effect of tectoridin on the Th17/Treg balance in psoriatic mice, a psoriasis model was established by applying 5% imiquimod cream to mice. The results indicated that tectoridin can ameliorate the pathological changes in the skin of psoriatic mice, inhibit abnormal proliferation, and reduce the inflammatory response. Specifically, tectoridin decreased the serum levels of TNF, IL-6, and IL-10, as well as the Th17/Treg ratio in peripheral blood [20].

Do discovered that isoswertisin, embinin, 6″-*O*-acetylembinin, 3″-*O*-acetylembinin, and iridin extracted from a 70% ethanol solution of *B. chinensis* significantly inhibited vascular smooth muscle cell (VSMC) proliferation, thereby effectively regulating the growth and proliferation of these cells [18]. Furthermore, the extracts of *B. chinensis* have been confirmed to exhibit antitussive, expectorant, and analgesic effects, as well as to enhance humoral immunity in healthy male ICR mice [19]. Fu et al. found that irisflorentin diminished LPS-stimulated dendritic cell (DC)-elicited allogeneic T-cell proliferation. Subsequently, treatment with irisflorentin clearly weakened 2,4-dinitro-1-fluorobenzene-induced delayed-type hypersensitivity. These findings suggest new insights into the role of irisflorentin as an immunotherapeutic adjuvant through its capability to modulate the properties of DCs [109]. Gary Ka et al. developed a screening platform whereby human embryonic kidney 293 cell (HEK293T) fibroblasts were transfected with a pTOPFLASH DNA construct, a screened tectoridin and extracts of Belamcandae Rhizoma were shown to significantly activate Wnt/catenin signaling in the assay of pTOPFLASH-transfected cells, which play a role in promoting hair growth in vibrissae hair follicles. Therefore, tectoridin, as well as the herbal extract of Belamcandae Rhizoma, possesses hair promoting activity, something which deserves further development [110]. The pharmacological activities, extract, model and results of *B. chinensis*. are summarized in Table 7.

Based on the above summary of the chemical constituents and pharmacological activities in *B. chinensis*, we now discuss the structure–activity relationships of the active compounds. Triterpenoids are primarily responsible for anti-inflammatory, antitumor, and antioxidant activities, with iridals being the main active triterpenoids. Comparison of compounds **1**–**16** revealed that only compounds **2**, **3**, and **14** exhibited significant anti-inflammatory activity. Unlike inactive compounds, these three lack a hydroxyl group at C-3 but possess a hydroxyl group at C-8, suggesting that hydroxylation at C-3 and C-8 is critical for activity. Furthermore, comparing compounds **2** and **14**, the latter showed superior activity, indicating that the configuration of the double bond between C-3 and C-7 also influences activity, with the *Z*-configuration being more favorable than the *E*-configuration. Overall analysis of compounds **17**, **23**, and **38** indicates that the presence of a spirocyclic structure at C-11 enhances activity. Iridal-type triterpenoids **2**–**4**, **10**, **14**, **16**, **17**, **19**, and **30** exhibited varying degrees of antitumor activity. Comparison of compounds **1**–**17** indicates that hydroxylation at C-4 slightly enhances activity compared with unsubstituted derivatives. In contrast, the presence of a long-chain acyl group at C-8 leads to loss of activity. Notably, compound **17** showed significantly stronger activity than other analogues, primarily due to the ether linkage between C-7 and C-9, which appears to be an activity-enhancing feature. Comparison of compounds **14** and **16** further indicates that a hydroxyl group at C-8 contributes to higher potency. Overall, compound **17** demonstrated the strongest activity among the active iridals (**2**–**4**, **10**, **14**, **16**, **17**, **19**), likely attributable to the cyclization between C-1 and C-7, which is proposed to be an activity-enhancing structural motif. Compounds **72–75** also exhibited certain antitumor activity. Comparison among compounds **73**–**75** revealed that substitution with a carbonyl group at C-3 leads to higher activity than hydroxyl substitution. Furthermore, the presence of a carboxyl group at C-18 confers superior activity compared with a hydroxymethyl group. Compounds **32**–**35** exhibited varying degrees of antioxidant activity. Compared with compound **30**, these active compounds lack methoxy groups, suggesting that methoxylation is detrimental to activity. Furthermore, the relative configuration of the aldehyde group at C-11 in compounds **30** and **31** is inverted when compared with that in the active analogues, indicating that the stereochemistry at C-11 is also a critical factor for activity. A detailed comparison of compounds **32**–**35** revealed that the *β*-orientation of the hydroxyl group at C-16 confers higher activity than the *α*-orientation. During comparison of compounds **39**–**50**, we found that only compounds **40** and **48** exhibited inhibitory activity against PTP1B, which revealed that the *E*-configuration of the double bond between C-3 and C-7 is essential for activity. Furthermore, the greater potency of compound **48** relative to **40** implies that the presence of an ethoxy group at C-26 enhances potency, suggesting that it functions as an activity-enhancing moiety. Separately, compounds **53** and **61** demonstrated kidney protective activity. Analysis of compounds **51**–**63** indicated that activity is retained when position C-12 is either unsubstituted or substituted with a carbonyl group.

Flavonoids exhibit diverse structures and demonstrate promising anti-inflammatory, antitumor, antioxidant, neuroprotective activities and anti-VSMC proliferation. Compounds **76**, **78**, **81**, **98**, **100**, **101**, **110**, **116**, **121**, **130**, and **139** displayed anti-inflammatory activity. A comparison of compounds **76**, **78**, **81**, **110**, and **113** revealed that the activity is positively correlated with the number of methoxy groups; however, the presence of a methoxy group at C-3′ reduces activity. Additionally, the dioxolane ring between C-6 and C-7 in compound 112 appears to diminish activity. Analysis of compounds **98**, **113**, and **130** suggests that the attachment position of the B-ring also influences activity, with linkage at C-2 conferring significantly higher activity than at C-3. Furthermore, comparison of compounds **121** and **139**–**141** indicated that a methoxy group at C-8 markedly enhances activity, suggesting that it serves as an activity-enhancing moiety. Isoflavonoids are the primary structural class responsible for the antitumor activity. A comparison of compounds **78**–**81** revealed that the activity depends on both the position and number of oxygen-containing groups on the B-ring. Activity is observed only when substitution occurs solely at C-4′ or when positions C-3′, C-4′, and C-5′ are all substituted. Analysis of compounds **98**–**101** indicated that the presence of a hydroxyl group at C-3′ leads to loss of activity, suggesting that it is a deactivating substituent. Combined with the analysis of the antioxidant activity of isoflavonoids, compounds **76**, **78**, **81**, 1**05**, **118**, and **121** exhibited certain activity. Evaluation of compounds **76**–**83** and **98**–**105** revealed that methoxy substitution at C-3′ abolishes activity, indicating that it acts as a deactivating group. The presence of a glycosyl group at the C-7 position of compound **105** significantly enhances its activity. Furthermore, activity is observed only when a single oxygen-containing substituent is present at C-4′ or when positions C-3′, C-4′, and C-5′ are all substituted. This substitution pattern is consistent with the structure–activity relationship described above for the antitumor activity of isoflavonoids, suggesting that the oxygenation pattern on the B-ring is a key factor influencing isoflavonoid activity. Compounds **78**, **81**, **89**, **98**, **101**, **105**, and **110** exhibited neuroprotective activity. A comprehensive analysis of compounds **76–110** confirms that the previously observed conclusion regarding B-ring oxygenation remains valid: activity depends on the presence of oxygen-containing substituents at either C-4′ alone or at C-3′, C-4′, and C-5′ simultaneously. Notably, compounds with monosubstitution at C-4′ showed slightly stronger activity than those substituted at all three positions. Furthermore, glycosylation at C-7 in compounds **98** and **105** contributed to a markedly enhanced activity when compared with other compounds. Based on the analysis of isoflavonoids with hypoglycemic activity (compounds **76**, **88**, **119**, and **120**), activity is observed only when an oxygen-containing substituent is present exclusively at the C-4′ position, a structural requirement distinct from those observed for antioxidant and neuroprotective activities. In contrast to its enhancing effect in other activity profiles, glycosylation at C-7 leads to reduced hypoglycemic activity. For compounds **122**, **123**, **144**, and **148**, a hydroxyl group at C-4′ confers stronger activity than a methoxy group at the same position, while a rhamnosyl moiety is associated with diminished efficacy.

Beyond their anti-inflammatory and antitumor activities, structurally diverse phenolic acids also demonstrate estrogen receptor modulating activity, bone metabolism regulation, and hypoglycemic effects. Among the mono-aromatic compounds **160**–**169**, only **160**, **161**, **166**, and **169** exhibited activity, suggesting that ester and aldehyde groups serve as key functional moieties, and that symmetrical substitution patterns also contribute to bioactivity. A comparison of compounds **171**–**174** revealed that the presence of a carbonyl group in the linker connecting the two benzene rings enhances activity, indicating that the carbonyl group functions as an activity-enhancing moiety. Moreover, shorter chain length between the aromatic rings correlates with improved efficacy.

**Figure 11:**
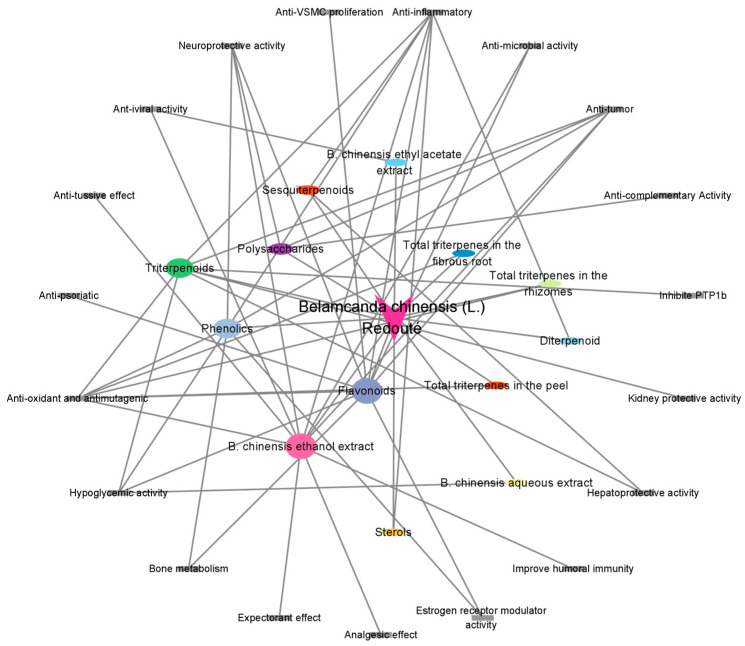
The correlation between active compounds/extracts and their pharmacological activities.

The correlation between active compounds/extracts and their pharmacological activities is summarized in Figure 11.

## 7. Toxicity

Despite the triterpenoids from *B. chinensis* possessing significant anti-inflammatory, anti-tumor, antioxidant, and kidney protective activities, they also pose a primary source of toxicity. Ito et al. found that hexane and ether extracts of *B. chinensis* exhibited ichthyotoxic activity. Specifically, 16-*O*-acetylisoiridogermanal, belachinal, and spiroiridal isolated from these extracts demonstrated highly ichthyotoxic activity against *Oryzias latipes*, with median tolerance limit (TLm) values ranging from 1.6 to 3.5 µg/mL after 24 h, which is comparable to buddledin B cytotoxicity on P-388 lymphocytes [5]. Song et al. isolated a novel dimeric triterpenoid, designated dibelamcandal A, from the rhizomes of *B. chinensis*. This compound exhibited significant molluscicidal activity against *Pomacea canaliculata*, with an LC_50_ value of 1.26 µg/mL and an LC_95_ value of 10.57 µg/mL [50]. Liu et al. isolated androsin from a 70% ethanol extract of *B. chinensis*, which was identified as a toxic component reported for the first time in this plant [73]. In the study conducted by Jahan et al., the toxicity of *B. chinensis* was evaluated using the *Brine Shrimp* lethality bioassay. The results indicated that the methanol and ethyl acetate soluble fractions demonstrated significant toxicity to *Brine Shrimp nauplii*, with LC_50_ values of 16.218 μg/mL and 0.048 μg/mL, respectively [106].

Documented in the *Ben Cao Gang Mu*, *B. chinensis* is characterized by a bitter taste and a cold property with mild toxicity. It is recorded that prolonged administration may result in physical weakness, and excessive consumption will induce diarrhea [32]. It is contraindicated in pregnant women to avoid potential adverse effects on the fetus. Processing reduces the toxicity of *B. chinensis*. Historically, it is documented that “*B. chinensis* is boiled with bamboo leaves from noon to midnight, then filtered and sun-dried” [115]. Zou et al. investigated the hepatosplenic toxicity of raw and processed *B. chinensis* with bamboo leaves by measuring serum biochemical indices AST, ALT, and TG in inflammation model rats. The results showed that the raw *B. chinensis* group exhibited significantly increased AST and ALT levels and decreased TG content. In contrast, the processed group demonstrated markedly reduced AST and ALT and increased TG compared with the raw group, indicating attenuated toxicity after processing with bamboo leaves [116]. In addition, Zuo et al. observed the effect of the extract of *B. chinensis* on digestive enzymes, amylase and gastrin in normal rats. The result is that the spleen index of rats fed with an extract of *B. chinensis* decreased, and that the rats showed decreased appetite, lethargy, weight loss and unformed stools, which indicated that the extract of *B. chinensis* had a certain hepatosplenic toxicity. Meanwhile, *B. chinensis* processed with rice swill can improve the conditions of decreased appetite and weight loss, while increasing serum amylase and gastrin levels. Rice-swilled processing of *B. chinensis* may mitigate its toxic effects by reinforcing the spleen [117]. A case of generalized muscle rigidity lasting for 6 h has been reported following self-administration of a soaked extract of *B. chinensis*. However, the underlying mechanism responsible for this case remains elusive. Further investigation is warranted to establish scientific treatment protocols for such poisoning, including whether muscle relaxants can be safely applied and any associated precautions [118].

In summary, while *B. chinensis* possesses valuable pharmacological properties, it is associated with mild yet non-negligible toxicity, as documented historically and confirmed by modern research. Scientific processing methods and adherence to appropriate usage guidelines are crucial to mitigating its toxic risks. Therefore, it is important to ensure its safe application and harness its therapeutic potential while minimizing potential hazards. The toxicity, extract, model and results of *B. chinensis* are summarized in Table 8.

## 8. Clinical Application and Product Development

### 8.1. Clinical Application

Clinically, *B. chinensis* is not typically used in isolation, rather, it is predominantly employed in conjunction with other medicinal herbs, among which Shegan Mahuang decoction is a classic prescription for the treatment of cold asthma. Modern clinical practice is mostly used to treat bronchial asthma, cough variant asthma, chronic bronchitis and chronic obstructive pulmonary disease and other respiratory diseases, with good clinical efficacy. To explore the effect of She Gan Ma Huang decoction combined with salbutamol in the treatment of elderly patients with an acute attack of bronchial asthma and its influence on respiratory function. Cai analyzed data from 94 elderly patients with acute bronchial asthma treated between November 2017 and November 2018. The results indicated that the combination of Shegan Mahuang decoction combined with salbutamol in the treatment of acute attacks of bronchial asthma in the elderly can significantly improve the clinical symptoms of patients, effectively adjust respiratory function, and offer a high level of safety [21]. Additionally, Fang et al. conducted a study on the effect of Shegan Mahuang decoction on pulmonary function, osteopontin (OPN) and TLR in patients with acute attack of bronchial asthma. They selected 86 patients from January 2016 to June 2020 for observation. The findings revealed that Shegan Mahuang decoction can effectively improve the treatment effect and lung function by modulating the serum hypersensitive C-reactive protein (hs-CRP), IL-6, IL-8, OPN and TLR in individuals suffering from acute bronchial asthma, confirming that the treatment is safe and reliable [22]. Li et al. conducted an analysis on the clinical effect of Shegan Mahuang decoction in patients with cough variant asthma. A total of 70 patients recruited from January 2020 to June 2022 were treated with either salmeterol ticasone inhalation powder spray or Shegan Mahuang decoction. The clinical outcomes of both treatment groups were subsequently compared. The findings indicated that Shegan Mahuang decoction significantly alleviates the clinical symptoms associated with cough variant asthma [23]. In a separate randomized controlled trial involving 70 elderly patients experiencing acute episodes of chronic bronchitis, participants were divided into two groups. The control group received conventional western medicine, while the study group was treated with Shegan Mahuang decoction in addition to the control treatment. Results demonstrated that Shegan Mahuang decoction effectively enhanced the treatment efficacy for patients suffering from acute attacks of chronic bronchitis while also reducing serum levels of IL-6, hs-CRP and procalcitonin (PCT) [24]. Zhu observed the clinical efficacy of Shegan Mahuang decoction in the treatment of the syndrome whereby cold fluid is retained in the lungs during acute exacerbation of chronic obstructive pulmonary disease (COPD). Both groups received standard treatment with Western medicine. In addition, the treatment group was given Shegan Mahuang decoction with oral administration. The results indicated that Shegan Mahuang decoction is effective in the treatment of the syndrome of cold fluid retention in the lungs during acute exacerbation of COPD [25]. Furthermore, Gancao Jiegeng Shegan decoction demonstrated significant therapeutic effects in the clinical treatment of acute simple pharyngitis, notably reducing patient pain and improving quality of life [119,120].

Numerous classical formulations are clinically used to treat various types of asthma and other respiratory diseases, with variations in efficacy and adverse effects. A network meta-analysis comparing nine classical prescriptions for asthma, in combination with conventional Western medicine, revealed that Shegan Mahuang decoction centered on *B. chinensis* demonstrated a superior overall response rate when compared with eight other formulations, including Yupingfeng San centered on Astragali Radix and Xiaoqinglong decoction centered on Ephedrae Herba and Ramulus Cinnamomi. Although its improvement in pulmonary function following treatment was lower than that of Yupingfeng San, it remained more effective than the other seven formulations, with no reported adverse events [121]. Furthermore, *B. chinensis* was ranked first in the frequency of use among formulations for treating cold asthma, outperforming other herbs such as *Pinellia ternata* (Thunb.) Ten. ex Breitenb. and Pinellia. [122].

To date, the preparation of a prescription containing *B. chinensis* as the primary ingredient is primarily utilized for treating respiratory diseases, showing significant therapeutic effect and minimal adverse reactions. This decoction can complement the combined treatment of Western medicine, highlighting its substantial research value and providing a model for the modern application of traditional medicine.

### 8.2. Product Development

Currently, there are 509 patents related to *B. chinensis* worldwide (https://www.lens.org/), with China holding the largest share at thirty-three percent. These patents primarily focus on applications in medicine, functional food, cosmetics, agriculture and other fields. Over ten proprietary Chinese medicines that incorporate *B. chinensis* are officially documented in the Chinese Pharmacopoeia (2020 Edition) (The Committee for the Pharmacopoeia of PR China, 2020) [35]. All of these include many TCM herbs or components demonstrating significant clinical efficacy. Notably, She Gan Kang Bing Du Zhu She Ye and She Gan Li Yan Kou Fu Ye are essential medications for the treatment of lung diseases in modern times, especially for the treatment of phlegm syndrome (https://www.nmpa.gov.cn/datasearch/search-result.html (accessed on 14 April 2025)). Proprietary Chinese medicines that include *B. chinensis* are shown in Table 9. Prudence is warranted with some patent medicines due to their complex components. For example, She Gan Kang Bing Du Zhu She Ye has been reported to cause allergic reactions, close monitoring is recommended during the initial administration [123]. In a rat model of COPD, She Ma Kou Fu Ye demonstrated therapeutic effects at medium and high dosage, with the high-dose group showing superior efficacy [124]. However, this preparation contains 8–14 mg of ephedrine per 15 mL, as well as refined honey as an excipient [35]. For COPD patients with tachyarrhythmia or diabetes, caution should be exercised regarding the dosage during use, and heart rate and blood sugar should be monitored during administration. During the course of Qing Yan Run Hou Wan, Qing Ge Wan, Qing Yan Li Ge Wan, and Gan Lu Xiao Du Wan, spicy, greasy, and rich foods are contraindicated. Among these, caution is advised regarding the use of Qing Yan Run Hou Wan and Qing Ge Wan in pregnant women and children [35], which is likely attributed to the documented toxicity of *Sophora tonkinensis* contained in their formulations. Despite the well-established efficacy and generally low toxicity of most Chinese medicines, clinical use should involve consideration of individual patient conditions, identification of contraindications, and appropriate dose adjustment to maximize treatment safety.

In the functional food industry, *B. chinensis* is combined with honeysuckle flowers, licorice, and other ingredients to produce a tea aimed at preventing and treating constipation in the elderly. The formulation consists of *Lonicerae Japonicae Flos* (30–50 parts), *B. chinensis* (10–30 parts), *Glycyrrhizae Radix et Rhizoma* (3–10 parts), along with *Chrysanthemi Flos*, *Canarii Fructus*, and Kudingcha. The mixture is decocted, filtered, and concentrated into an extract. Subsequently, honey (10–50 parts) and spring water (200–600 parts) are added. The final product is obtained after a sterilization process. The efficacy was assessed using a mouse model of constipation. Results indicated that all three tested doses of the formulation significantly enhanced intestinal motility and reduced the time to first defecation. This tea effectively clears heat and toxins, relieves constipation, and is suitable for long-term consumption by the elderly [125]. A healthcare tea bag incorporating traditional Chinese medicines has also been developed, containing 10–30 parts of *Elsholtzia ciliate*, 10–30 parts of *B. chinensis*, 10–30 parts of *Alpinia katsumadai* seeds, 5–10 parts of *Osmanthus fragrans* flowers, and 5–10 parts of *Asarum sieboldii*. The weighed herbal materials are dried, pulverized, sieved, and packed into tea bags. In a clinical observation involving 270 patients with oral malodor, the preparation was administered as a tea infused with hot water after meals. The results demonstrated an improvement rate of 91.11% in halitosis symptoms, indicating its effectiveness in eliminating bad breath with regular consumption [126].

In the cosmetics industry, *B. chinensis* is valued for its potent anti-inflammatory, antioxidant, and antibacterial activities. One cosmetic composition contains 0.05–10.0 wt% of a *B. chinensis* extract, 0.025–5.0 wt% of vitamin C, and/or 0.025–5.0 wt% of vitamin E. The *B. chinensis* extract was prepared using a mixed water–alcohol solvent, followed by the addition of EtOAc, CHCl_3_, or CH_2_Cl_2_ and water to separate the phases. The organic solvent fraction was collected and dried under reduced pressure. Bioactivity assays confirmed that this composition inhibits iNOS expression in skin cells, particularly keratinocytes and fibroblasts, thereby suppressing NO production and effectively cleansing the skin [127]. Additionally, a composition for hair dyeing and scalp care contains 0.01–30 wt% of an alcohol extract or a hot water extract of B. chinensis. The alcohol extraction involves heating at 30–50 °C for 8–12 h, while the hot water extraction is performed at 60–90 °C for 10–15 h. Testing has shown that this composition promotes the coordinated synthesis of several proteins and enzymes involved in the production of elastic fibers, thereby improving skin elasticity. Consequently, the *B. chinensis* extract utilized in protective hair dyes for hair and scalp enhances hair gloss without causing harm to the human body and alleviates issues related to hair and scalp irritation [128].

In agriculture, *B. chinensis* serves as a key component in a natural bactericide for preventing and treating peach brown rot. One such fungicide is composed of the following components by weight: *B. chinensis* (0.5–1.5 parts), *Momordicae Charantiae Fructus*, *Phragmitis Rhizoma*, *Puerariae Thomsonii Radix* (4–6 parts), *Aristolochiae Fructus*, *Atractylodis Macrocephalae Rhizoma* (2–4 parts), *Polygoni Cuspidati Rhizoma*, *Bruceae Fructus*, *Pogostemonis Herba* (1–3 parts), and *Pyrolae Herba* (0.5–1.5 parts). The raw materials are pulverized, sieved, and thoroughly mixed. This mixture is then extracted with 5–10 volumes of a 95% ethanol aqueous solution at 50–70 °C for 2–4 h. After filtration, the residue undergoes a second extraction under the same conditions. The combined filtrates are concentrated until the alcohol odor is eliminated, and the resulting concentrate is dissolved in water to obtain the final fungicide preparation. In mycelial growth rate assays, this fungicide demonstrated high efficacy against *Monilinia fructicola*, achieving an inhibition rate greater than 86% [129]. Beyond its widespread use in pharmaceuticals, *B. chinensis* has been utilized in diverse product forms across different regions. In southern China, it serves as a key ingredient in functional foods and agricultural products but constitutes a minor component in agricultural products. Meanwhile, South Korea has primarily explored the potential of its extract in the cosmetics industry. The preparation methods vary significantly depending on the intended application: functional teas typically employ simple decoction, whereas cosmetics and agricultural fungicides require extraction with organic solvents to isolate the active components.

Overall, *B. chinensis* not only demonstrates a significant role in the clinical treatment of respiratory system diseases, but also shows considerable potential for growth in the development of functional foods, cosmetics and agricultural products.

## 9. Conclusions and Prospect

This review systematically summarizes recent advances in traditional applications, phytochemistry, pharmacology, toxicology and clinical application of *B. chinensis*, and the structure–activity relationships of the active compounds were discussed. Widely utilized in traditional Chinese medicine for treating throat disorders, this plant has yielded 228 identified chemical components, including 75 triterpenoids, 84 flavonoids,40 phenolics, and 29 other compounds, with triterpenoids and flavonoids representing the majority of newly identified constituents. Pharmacological investigations reveal that extracts and isolated compounds from *B. chinensis* exhibit anti-inflammatory, antitumor, antioxidant, neuroprotective, and hypoglycemic activities in vitro and in vivo. Moreover, a variety of Chinese patent medicines containing *B. chinensis* have been marked in China, demonstrating its significant application value in modern medicine, as well as the key role it plays in food, cosmetics and other fields.

However, there are still limitations in the existing research on *B. chinensis*. Firstly, most of the research on the phytochemical composition of *B. chinensis* have concentrated on the rhizomes, likely due to their status as the active part. It is noteworthy that iridal-type triterpenoids with a novel carbon framework have been identified in the dried seeds, suggesting that other parts of the plant warrant greater attention to potentially discover more structurally unique compounds and maximize the utilization of *B. chinensis*. Secondly, pharmacological activity studies have predominantly focused on in vitro evaluations, while in vivo studies and mechanistic investigations of its pharmacological effects remain limited. Conducting in vivo activity studies is crucial for providing data support for clinical research. Thirdly, *B. chinensis* contains a variety of active ingredients, and the main components along with their interactions require in depth exploration to systematically elucidate the primary active ingredients and the mechanism of action of *B. chinensis.* Additionally, *B. chinensis* is frequently used in combination with other drugs to treat related diseases; however, its interaction needs further investigation, and the risk of drug interactions poses a potential concern. Therefore, a systematic study on the interaction between *B. chinensis* and other commonly used drugs is necessary to provide guidance for the clinical rational use of drugs in clinical settings.

All in all, as a traditional medicinal plant, *B. chinensis* has a long history of use both domestically and internationally, characterized by its wide distribution and abundant resources. Existing studies have demonstrated that *B. chinensis* possesses significant application value not only in the prevention and treatment of diseases, but also in the preparation of functional food and cosmetics and the development of agricultural products. The research of natural products also shows that *B. chinensis* has the potential to develop compounds with novel structures and excellent activity biological activity. Consequently, this paper summarizes the chemical composition, traditional use, pharmacological activities toxicity and clinical application of *B. chinensis*, providing valuable references for its comprehensive utilization.

## Figures and Tables

**Table 6:** Isolation and identification of polysaccharides from *B. chinensis*.

NO.	Name	Monosaccharide Residues	Parts	References
**1**	BCP-A1	*β*-D-Man*p*-(1→, *β*-D-Glc*p*-(1→, →4)-*α*-D-Gal*p*-(1→ and →3,4)-*β*-D-Gal*p*-(1→	Rhizomes	[13]
**2**	BCP-B1	→5)-*α*-L-Ara*f*-(1→, *β*-D-Man*p*-(1→, *β*-D-Glc*p*-(1→, →4)-*α*-D-Glc*p*, →4)-*α*-D-Gal*p*-(1→, →4)-*α*-D-Gal*p*A-(1→ and →3,4)-*β*-D-Gal*p*-(1→	Rhizomes	[13]
**3**	BCP50-2	→3,5)-*α*-L-Ara*f*-(1→, →4)-*β*-D-Gal*p*-(1→, →4,6)-*β*-D-Gal*p*-(1→, →3)-*α*-L-Galp-(1→	Rhizomes	[10]
**4**	BCP80-2	t-*α*-Ara*f*-(1→, →3,5)-*α*-Ara*f*-(1→, →5)-*α*-Ara*f*-(1→, →4)-*β*-Xyl*p*-(1→, →3)-*α*-Rha*p*-(1→, →4)-*β*-Manp-(1→, t-*β*-Glc*p*-(1→, →6)-*α*-Glc*p*-(1→, t-*β*-Gal*p*- (1→, and→3)-*α*-Gal*p*-(1→	Rhizomes	[11]
**5**	BCP30-1a	Man*p*-(1→→5)-Ara*f*-(1→ Gal*p*-(1→→3)-Gal*p* (or GalA*p*)-(1→→4)-Glc*p*-(1→→3,6)-Gal*p*-(1→	Rhizomes	[12]
**6**	BCP50-1a	Man*p*-(1→→5)-Ara*f*-(1→→3)-Gal*p* (or GalA*p*)-(1→→4)- -Glc*p*-(1→→3,6)-Gal*p*-(1→	Rhizomes	[12]
**7**	BCP70-1a	Man*p*-(1→→5)-Ara*f*-(1→→3)-Gal*p* (or GalA*p*)-(1→→4)- -Glc*p*-(1→→3,6)-Gal*p*-(1→	Rhizomes	[12]
**8**	BCP90-1a	Man*p*-(1→→5)-Ara*f*-(1→→3)-Gal*p* (or GalA*p*)-(1→→4)- -Glc*p*-(1→→3,6)-Gal*p*-(1→	Rhizomes	[12]

**Table 7:** The pharmacological activities, extract, model and results of *B. chinensis*. are summarized.

Pharmacological Effects	Extracts/Compounds	Type	Models/Methods	Effects /Mechanisms	References
Anti-inflammatory					
	Isoiridogermanal	In vitro	HNE enzyme	IC_50_ = 14.4 ± 0.3 µM	[43]
	Iridobelamal A	In vitro	HNE enzyme	IC_50_ = 27.0 ± 0.6 µM	[43]
	iridobelamal B	In vitro	HNE enzyme	IC_50_ = 6.8 ± 0.3 µM	[43]
	Isoiridogermanal	In vitro	RAW 264.7 cells	iNOS ↓, IL-1β ↓, TNF ↓	[43]
	Iridobelamal A	In vitro	RAW 264.7 cells	iNOS ↓, IL-1β ↓, TNF ↓	[43]
	Dehydrocostus lactone	In vitro	RAW 264.7 cells	NO: IC_50_ = 8.8 µM	[51]
	Iristectorigenin B	In vitro	RAW 264.7 cells	NO: IC_50_ = 52.6 µM	[51]
	Tectorigenin	In vitro	RAW 264.7 cells	NO: IC_50_ = 31.6 µM	[51]
	Irisflorentin	In vitro	RAW 264.7 cells	NO: IC_50_ = 51.9 µM	[51]
	Irigenin	In vitro	RAW 264.7 cells	NO: IC_50_ = 26.0 µM	[51]
	5-*O*-Demethylnobiletin	In vitro	RAW 264.7 cells	NO: IC_50_ = 37.0 µM	[51]
	Vitexilactone	In vitro	RAW 264.7 cells	NO: IC_50_ = 43.6 µM	[51]
	Vanillin	In vitro	RAW 264.7 cells	NO: IC_50_ = 18.0 µM	[51]
	Apocynin	In vitro	RAW 264.7 cells	NO: IC_50_ = 45.0 µM	[51]
	Tectoridin A	In vitro	SW480 human colon cancer cells	NF-κB inhibitory rates = 53.71%	[63]
	Nigricin A	In vitro	SW480 human colon cancer cells	NF-κB inhibitory rates = 57.68%	[63]
	Naringenin	In vitro	SW480 human colon cancer cells	NF-κB inhibitory rates = 88.71%	[63]
	Irigenin	In vitro	RAW 264.7 cells	iNOS ↓, COX-2 ↓, NF-κB ↓	[80]
	3′,5,7-Trihydroxy-8,4′-dimethoxyisoflavone	In vitro	RAW 264.7 cells	COX-2: IC_50_ = 4.20 µM	[56]
	16-*O*-Acetyliridobelamal A	In vitro	RAW 264.7 cells	NO: IC_50_ = 3.53 ± 0.24 µM, IL-6 ↓	[16]
	16-*O*-Acetyl-iso-iridogermanal	In vitro	RAW 264.7 cells	NO: IC_50_ = 4.13 ± 0.05 µM	[16]
	(6*R*, 10*S*, 11*S*, 14*S*, 26*R*)-(+)-29-Acetoxy-14,15-Dihydro-26-hydroxyspiroirida-15(28)-16-diena	In vitro	RAW 264.7 cells	NO: IC_50_ = 0.56 ± 0.04 µM	[16]
	Belamcanoxide B	In vitro	RAW 264.7 cells	NO: IC_50_ = 23.0 ± 5.00 µM	[16]
	1, 4-Dimethoxybenzene	In vitro	RAW 264.7 cells	NO: IC_50_ = 12.7 ± 1.30 µM	[16]
	(22*E*)-5*α*, 8*α*-Epidioxyergosta-6, 22-dien-3*β*-ol	In vitro	RAW 264.7 cells	NO: IC_50_ = 14.6 ± 2.20 µM	[16]
	16-*O*-Acetyliridobelamal A	In vitro	RAW 264.7 cells	Inhibition of IL-6 activation: 86.49 ± 0.45%	[16]
	Stigmasta-4,6,8(14), 22-tetraen-3-one	In vitro	RAW 264.7 cells	Inhibition of IL-6 activation: 65.55 ± 4.99%	[16]
	Irigenin	In vitro	HCE-2 cells	IL-1β ↓, TNF ↓, GSDMD ↓, IL-18 ↓, caspase-1 ↓, HAS2 and HAS3 ↑	[82]
	Iristectorin A	In vitro	RAW 264.7 cells	NO ↓, TNF ↓, IL-β ↓, IL-6 ↓, iNOS ↓, COX-2 ↓	[81]
	Iristectorin B	In vitro	RAW 264.7 cells	NO ↓, TNF ↓, IL-1β ↓, IL-6 ↓, iNOS ↓, COX-2 ↓	[81]
	Mangiferin	In vivo	Male Wistar rats	TNF ↓, IL-17 ↓	[83]
	*B. chinensis* extract	In vivo	180~220 g Wistar healthy mice	The rat paw swelling ↓	[19]
	*B. chinensis* extract	In vivo	18~22 g healthy male ICR mice	The ear swelling induced by xylene ↓	[19]
	*B. chinensis* extract	In vivo	RSV-infected guinea pig model	IL-4 ↓, IFN-γ ↑, IL-4/IFN-γ ↓	[84]
	*B. chinensis* ethanol extract	In vitro	RAW 264.7 cells	NO ↓, IL-6 ↓, TNF ↓	[111]
	*B. chinensis* ethanol extract	In vitro	RAW 264.7 cells	Inhibitory effect of xylene on ear swelling in mice at 28.96% and 37.54%, NO ↓, iNOS ↓ TNF ↓, IL⁃1β ↓, IL⁃6 ↓	[85]
	*B. chinensis* extract	In vivo	C57BL/6J female mice aged 3 weeks	IL-6 ↓, IL-12 ↓, IL-1β ↓, NO ↓, MCP-1 ↓, IL-10 ↑, IL-13 ↑	[86]
Anti-tumor					
	Belamcanoxide B	In vitro	HCT-116 and MCF-7 cell lines	IC_50_ = 5.58, 3.35 μM	[40]
	16-*O*-Acetylisoiridogermanal	In vitro	HepG2 and MCF-7 cell lines	IC_50_ = 7.66, 6.43 μM	[40]
	3-*O*-Acetyliridobelamal A	In vitro	HCT-116 and HepG2 cell lines	IC_50_ = 8.71, 7.22 μM	[40]
	Belamchinenin A	In vitro	NCI-H1650 HepG2, BGC 823, HCT-116 and MCF-7 cell lines	IC_50_ = 2.48, 2.55, 4.47, 2.29, and 2.85 μM	[44]
	Belamcanoxide A	In vitro	HepG2, BGC 823, NCI-H1650, and HCT-116	IC_50_ = 6.17, 3.26, 5.84 and 7.53 μM	[41]
	Iridobelamal A	In vitro	BGC-823 and MCF-7	IC_50_ = 5.12 and 8.23 μM	[41]
	Isoiridogermanal	In vitro	BGC 823, NCI-H1650, and HCT-116	IC_50_ = 5.61, 8.63, 7.62 μM	[41]
	Iridal	In vitro	HepG2, BGC-823, NCI-H1650	IC_50_ = 5.19, 4.76, 8.51 μM	[41]
	Kampferol	In vitro	MGC-803, Bcap-37, MCF-7, PC3, NIH3T3	Inhibitory Rate: 58.2 ± 3.0, 51.2 ± 8.1, 39.2 ± 6.8, 46.1 ± 5.9, 11.1 ± 6.7	[49]
	Ursolic acid	In vitro	MGC-803, Bcap-37, MCF-7, PC3, NIH3T3	Inhibitory Rate: 51.7 ± 5.6, 48.4 ± 5.9, 49.4 ± 4.1, 57.7 ± 1.9, 21.7 ± 4.9	[49]
	Betulin	In vitro	MGC-803, Bcap-37, MCF-7, PC3, NIH3T3	Inhibitory Rate: 43.7 ± 6.7, 53.2 ± 3.2, 53.2 ± 5.4, 17.3 ± 5.2, 33.5 ± 7.1	[49]
	Betulonic acid	In vitro	MGC-803, Bcap-37, MCF-7, PC3, NIH3T3	Inhibitory rate: 68.1 ± 2.6, 44.9 ± 2.9, 56.1 ± 4.4, 52.4 ± 4.2, 22.1 ± 6.2	[49]
	Betulone	In vitro	MGC-803, Bcap-37, MCF-7, PC3, NIH3T3	Inhibitory rate: 52.2 ± 5.3, 54.2 ± 2.2, 64.7 ± 7.3, 52.3 ± 3.3, 36.3 ± 7.1	[49]
	Isoiridogermanal	In vitro	BT549, 4T1, MDA-MB-468, MDA-MB-231, and MCF7 cell lines	IC_50_ = 29.16 μM, 13.51 μM, 12.76 μM, 13.02 μM, 59.80 μM, 39.83 μM	[49]
	4′-*O*-Methylnyasol	In vitro	K562 human leukemia cell line	IC_50_ = 4.20 μM.	[63]
	Tectorigenin, irigenin	In vitro	LNCaP cell	PDEF, PSA, IGF-1 receptor mRNA expression and hTERT mRNA expression and telomerase activity ↓	[6]
	Tectorigenin	In vitro	Osteosarcoma cell	Proliferation migration and invasion of cell ↓, the expression of cleaved ↓, caspase3 ↓, the expression of MMP1, MMP2, and MMP9 ↓	[93]
	Isoiridogermanal	In vitro	MDA-MB-468 cell lines	IC_50_ = 15.74 ± 1.04 μM, cell proliferate and migration ↓, cell cycle was arrested at G1 phase, mitochondrial membrane potential ↓, ROS ↑	[42]
	Isoiridogermanal B	In vitro	MDA-MB-468 cell lines	IC_50_ = 26.97 ± 2.68 μM, cell proliferate and migration ↓, cell cycle was arrested at G1 phase, mitochondrial membrane potential ↓, ROS ↑	[42]
	Tectoridin	In vitro	H299 and A549 cells	ROS ↑, inhibit PI3K/AKT pathway, p-AKT ↓, E-cadherin ↑, EMT occurrence ↓	[90]
	Tectoridin	In vitro	HGC-27 cells	Inhibited proliferation of HGC-27 cells, expression of Beclin-1, p53 and BAX ↑, apoptosis of HGC-27 cells ↑, expression of Bcl-2 proteins ↓, p-PI3K p-Akt, p-mTOR ↓	[92]
	BCP80-2	In vitro	HepG2 cells	Suppressing the FAK signaling pathway, CD40, CD86, CD80, and MHC-II ↑	[11]
	*B. chinensis* ethanol extract	In vitro	H460 cells	MicroRNA-21 expression levels ↓	[91]
	*B. chinensis* extract	In vivo	Six-week-old male athymic nude BALB/c-nu mice received LNCaP cells	Tumor volume ↓, delayed onset of tumor growth in treated mice, PSA ↓	[6]
	*B. chinensis* extract	In vivo	AOM/DSS mouse model	STAT3 protein levels ↓, IL6 ↓	[88]
	*B. chinensis* extract	In vivo	AOM/DSS mouse model	MAPK ↓, PI3K ↓	[89]
	BCE	In vitro	LNCaP cells	Expression of the AR, PDEF, NKX3.1 and PSA ↓, AR protein and PSA secretion ↓, ER-β expression ↑	[87]
Antioxidant and antimutagenic					
	Belamchinenin C	In vitro	Fe^2+^/cysteine-induced liver microsoma	Inhibitory activities: 51.95%	[45]
	Belamchinenin D	In vitro	Fe^2+^/cysteine-induced liver microsoma	Inhibitory activities: 54.52%	[45]
	Belamchinenin E	In vitro	Fe^2+^/cysteine-induced liver microsoma	Inhibitory activities: 33.76%	[45]
	Belamchinenin F	In vitro	Fe^2+^/cysteine-induced liver microsoma	Inhibitory activities: 45.98%	[45]
	Belamcanoside A	In vitro	HepG2 cells	The expression of HMGCR, SQLE ↓, LDLR ↑, SORT1 ↓	[55]
	Belamcanoside B	In vitro	HepG2 cells	Regulate the expression of HMGCR, SQLE ↓	[55]
	Dalspinin	In vitro	DPPH assay	IC_50_ = 10.44 µM	[56]
	Iristectorigenin B	In vitro	ABTS assay	IC_50_ = 13.49 µM	[56]
	3′,5,7-Trihydroxy-8,4′-dimethoxyisoflavone	In vitro	FRAP	4.77 mM Fe^2+^	[56]
	Dichotomintin	In vitro	DPPH	IC_50_ = 190.1 mg/mL	[7]
	Irigenin	In vitro	DPPH	IC_50_ = 76.6 mg/mL	[7]
	Tectorigenin	In vitro	DPPH	IC_50_ = 28.5 mg/mL	[7]
	Iridin	In vitro	DPPH	IC_50_ = 10.2 mg/mL	[7]
	Total triterpenes in the peel	In vitro	0.1% FeCl_3_ solution, DPPH, ABTS working Solution	Total reducing power ↑, DPPH: IC_50_ = 0.4589 mg/mL, ABTS^+^ = 0.3273 mg/mL	[94]
	Total triterpenes in the rhizome	In vitro	0.1% FeCl_3_ solution, DPPH, ABTS working Solution	total reducing power ↑, DPPH: IC_50_ = 0.8396 mg/mL, ABTS^+^ = 0.3892 mg/mL	[94]
	Total triterpenes in the fibrous root	In vitro	0.1% FeCl_3_ solution, DPPH, ABTS working Solution	total reducing power ↑, DPPH: IC_50_ = 4.058 mg/mL, ABTS^+^ = 0.3368 mg/mL	[94]
	Total triterpenes in the peel	In vivo	Obese model mice	Weight loss in obese mice, Lee’s index ↓, fat in the liver ↓, epididymis fat weight index ↓, LDL-c ↓, HDL-C ↑, LEP ↓, GSH ↑, SOD ↑, MDA ↓, improve liver cells	[94]
	BCPT	In vitro	DPPH, ABTS^+^	Scavenging rates: 78.92%, 96.08%	[112]
	BCRT	In vitro	DPPH, ABTS^+^	Scavenging rates: 58.01%, 94.32%	[112]
	*B. chinensis* extract	In vitro	*Salmonella* TA98 and TA100	The number of spontaneous revertants ↓	[113]
	*B. chinensis* extract	In vitro	DPPH	EC_50_ = 11.7~174.2 µg/mL	[113]
	*B. chinensis* extract	In vitro	Phosphomolybdenum assay	P-Mo AAE = 0.16~1.48 g/g d.w.	[113]
	*B. chinensis* extract	In vitro	Linoleic acid	Maximum inhibition % of linoleic acid peroxidation: 22.24~99.48 µg/mL	[113]
Neuroprotective activity					
	Total extract of *B. chinensis*	In vitro	PC12 cells damaged by MPP^+^	Cell survival rate ↑	[9]
	Tectoridin	In vitro	Lactate dehydrogenase	Enhancement factor: 7.17~10.62%	[9]
	Iridin	In vitro	Lactate dehydrogenase	Enhancement factor: 8.02~12.44%	[9]
	Tectorigenin	In vitro	Lactate dehydrogenase	Enhancement factor: 25.99~33.72%	[9]
	Irisflorentin	In vitro	Lactate dehydrogenase	Enhancement factor: 49.82~72.75%	[9]
	Irigenin	In vitro	Lactate dehydrogenase	Enhancement factor: 23.02~33.49%	[9]
	Piceatannol	In vitro	AchE, BChE	AChE: IC_50_ = 53.42 ± 2.22 μg/mL, BChE: IC_50_ = 18.20 ± 0.89 μg/mL	[62]
	Irilin D	In vitro	BChE	IC_50_ = 109.53 ± 6.02 μg/mL	[62]
	Resveratrol	In vitro	BChE	IC_50_ = 78.07 ± 4.24 μg/mL	[62]
	Iristectorin B	In vitro	OGD PC12 cells	Increased cell survival, Ca^2+^, LDH, ROS ↓, adjust HMOX1, TFR1, SLC3A2	[95]
	Tectorigenin	In vitro	NT2/D1 cell	HRE-driven luciferase activity ↑, induced the expression of HIF-1*α* protein, and EPO mRNA	[98]
	Tectorigenin	In vitro	Rat cortical neurons	Induced the expression of HIF-1α protein	[98]
	Irigenin	In vitro	Mouse microglia BV-2 cells	Suppressed MPP^+^-induced viability reduction, activated the Keap1/Nrf2 pathway in MPP^+^-induced BV-2 cells	[99]
	BCP50-1a	In vitro	OGD PC12 cells	Cell survival rate ↑	[12]
	BCP70-1a	In vitro	OGD PC12 cells	Cell survival rate ↑	[12]
	BCP90-1a	In vitro	OGD PC12 cells	Cell survival rate ↑	[12]
Hypoglycemic activity					
	Swertisin	In vitro	*α*-glucosidase	IC_50_ = 119 µg/mL	[53]
	2″-*O*-Rhamnosylswertisin	In vitro	*α*-glucosidase	IC_50_ = 333 µg/mL	[53]
	Genistein	In vitro	*α*-glucosidase	IC_50_ = 74 µg/mL	[53]
	Genistin	In vitro	*α*-glucosidase	IC_50_ = 83 µg/mL	[53]
	Mangiferin	In vitro	*α*-glucosidase	IC_50_ = 112 µg/mL	[53]
	Daidzin	In vitro	*α*-glucosidase	IC_50_ = 97 µg/mL	[53]
	19*α*-Trihydroxy-28-norurs-12-ene	In vitro	*α*-glucosidase	IC_50_ = 84.90 ± 3.71 μM	[16]
	6-Hydroxybiochanin A	In vitro	*α*-glucosidase	IC_50_ = 61.85 ± 2.12 μM	[16]
	5,4′-Dihydroxy-7,3′-Dimethoxyflavanone	In vitro	*α*-glucosidase	IC_50_ = 93.75 ± 0.65 μM	[16]
	5-Hydroxy-7,3′,4′-Trimethoxyflavanone	In vitro	*α*-glucosidase	IC_50_ = 164.01 ± 1.54 μM	[16]
	*trans*-Resveratrol	In vitro	*α*-glucosidase	IC_50_ = 24.49 ± 0.22 μM	[16]
	Tectorigenin	In vitro	*α*-glucosidase	Enhancement factor ↑	[9]
	Irigenin	In vitro	*α*-glucosidase	Enhancement factor ↑	[9]
	Iristectorigenin A	In vitro	*α*-glucosidase	Enhancement factor ↑	[9]
	Irisflorentin	In vitro	*α*-glucosidase	Enhancement factor ↑	[9]
	Tectoridin	In vivo	Streptozotocin-induced diabetic rats	Inhibitory potency of aldose reductase: IC_50_ = 1.08 µM, sorbitol accumulation in the lens, sciatic nerves and red blood cells ↓	[101]
	Tectorigenin	In vivo	Streptozotocin-induced diabetic rats	Inhibitory potency of aldose reductase: IC_50_ = 1.12 µM, sorbitol accumulation in the lens, sciatic nerves and red blood cells ↓	[101]
	BCL	In vitro	*α*-glucosidase	IC_50_ = 800 µg/mL	[53]
	BCL	In vivo	Male Kunming mice	The blood glucose levels in normal mice at 1 h after starch intake ↓	[53]
	BIF	In vitro	*α*-glucosidase	IC_50_ = 500 µg/mL	[53]
	BIF	In vivo	Male Kunming mice	The blood glucose levels in normal mice at 1 h after starch intake ↓	[53]
	BCLE F1	In vivo	KK-A^y^ mice	SOD ↑, MDA ↓, LDL-c ↓, TG ↓, HDL-c ↑	[15]
	BCLE F2	In vivo	KK-A^y^ mice	The body weight gain of obese mice ↓, the liver weight and index ↓, SOD ↑, MDA ↓, TC ↓, TG ↓, LDL-c ↓	[15]
	BCLE F2	In vivo	KK-A^y^ mice	FBG, AUC, GSP, LDH and insulin ↓, hepatic G6Pase and PEPCK ↓, inhibited GSK-3β, enhanced liver glycogen, PPAR γ ↑	[15]
	BCLE F1	In vivo	KK-A^y^ mice	Cell degeneration ↓, pathological tissue injury ↓	[15]
Hepatoprotective activity					
	Iristectorigenin B	In vitro	HEK 293 cells	Induced the transactivation of both LXR-α, 540% at 20 μM and LXR-β, 331% at 20 μM	[102]
	Iristectorigenin B	In vitro	RAW 264.7 cells	Cholesterol efflux by inducing ABCA1 and ABCG1 ↑, cellular cholesterol concentration ↓	[102]
	Belchinoid A	In vitro	HepG2 cells	TG ↓	[16]
	Belchinoid B	In vitro	HepG2 cells	TG ↓	[16]
	3-*O*-Capryloyl-16-*O*-acetylisoiridogermanal	In vitro	HepG2 cells	TG ↓	[16]
	16-*O*-Acetyliridobelamal A	In vitro	HepG2 cells	TG ↓	[16]
	Anhydrobelachinal	In vitro	HepG2 cells	TG ↓	[16]
	Tectorigenin	In vivo	CCl_4_-intoxicated rats	MDA ↓, AST and ALT level ↓, SOD, catalase, and GSH-px enzyme ↑	[8]
	Tectoridin	In vivo	CCl_4_-intoxicated rats	MDA ↓, AST and ALT level ↓, SOD, catalase, and GSH-px enzyme ↑	[8]
	BRC-NCs	In vivo	Acute liver injury model of mice induced by carbon tetrachloride (CCL_4_)	ALT, AST, DBIL, IBIL in serum and MDA in liver homogenate ↓, SOD ↑, reduce the liver tissue damage	[103]
Anti-microbial activity					
	Tectorigenin	In vitro	Dermatophytes	MIC ranging from 3.12–6.25 mL	[106]
	Irisflorentin	In vitro	*Escherichia coli*	Up-regulate the expression of phagocytic receptors SR-A1, enhance the ability of macrophages to phagocytose pathogens.	[105]
	*B. chinensis* extract	In vitro	*Staphylococcus aureus*	MIC = 0.0625 g/mL	[19]
	*B. chinensis* extract	In vitro	*Streptococcus pneumoniae*	MIC = 0.0156 g/mL	[19]
	*B. chinensis* extract	In vitro	*Escherichia coli*	MIC = 0.25 g/mL	[19]
	*B. chinensis* extract	In vitro	*Pseudomonas aeruginosa*	MIC = 0.0312 g/mL	[19]
	*B. chinensis* extract	In vitro	*Streptococcus agalactiae*	MIC = 0.0156 g/mL	[19]
	*B. chinensis* extract	In vitro	*Streptococcus pyogenes*	MIC = 0.0156 g/mL	[19]
	*B. chinensis* extract	In vitro	*Shigella dysenteriae*	MIC = 0.0625 g/mL	[19]
	*B. chinensis* extract	In vivo	Mice induced by intraperitoneal injection of *Staphylococcus aureus*	Mouse mortality caused by staphylococcus aureus yeast suspension ↓	[19]
	*B. chinensis* extract	In vitro	*S. aureus*, *S. pneumoniae*, *B. coli*, *P. aeruginosa*, *S. agalactiae*, *M. scarlatinae* and *S. dysenteriae*	MIC = 0.0625, 0.0156, 0.2500, 0.0312, 0.0156, 0.0156, 0.0625 g/mL	[104]
Antiviral activity					
	*B. chinensis* extract	In vitro	Influenza virus FM1 in MDCK cells	EC_50_ = 122.17 µg/mL, TC_50_/EC_50_ = 19.16	[19]
	*B. chinensis* extract	In vitro	Respiratory syncytial virus in Hela Cells	EC_50_ = 119.16 µg/mL TI = 34.94	[19]
	*B. chinensis* ethyl acetate extract	In vitro	Influenza virus sub-A murine lung adapted strain FM1	Reduce virus titer by more than 2 logs	[114]
	*B. chinensis* ethyl acetate extract	In vivo	Virus nasal infected Kunming mice	Death rate ↓, mean survival time ↑	[114]
Bone metabolism					
	Mangiferin	In vivo	ovariectomized female Wistar rats	BCTX ↓, BMD ↑	[107]
	Irilin D	In vivo	A mouse model of LPS-induced bone loss	Blocked osteoclastogenesis, disrupted RANKL-induced activation of mitogen-activated protein kinases and nuclear factor-κB	[108]
	Belamchinoside A	In vitro	RAW 264.7 cells	RANKL-induced osteoclast formation ↓, RANKL-induced TRAP-positive multinucleated osteoclast formation ↓	[76]
	Irilin D	In vitro	RAW 264.7 cells	RANKL-induced osteoclast formation ↓, RANKL-induced TRAP-positive multinucleated osteoclast formation ↓	[76]
Estrogen receptor modulator activity					
	Resveratrol	In vitro	MCF-7, T47D cell lines	MCF-7: EqE_10_ = 1.6 µM T47D: EqE_10_ = 0.03 µM	[58]
	Iriflophenone	In vitro	MCF-7, T47D cell lines	MCF-7: EqE_10_ = 0.7 µM, EqE_100_ = 6.8 µM T47D: EqE_10_ = 4.9 µM	[58]
	Tectorigenin	In vitro	MCF-7, T47D cell lines	MCF-7: EqE_10_ = 0.3 µM, EqE_100_ = 1.0 µM T47D: EqE_10_ = 0.04 µM, EqE_100_ = 0.5 µM	[58]
	Tectoridin	In vitro	MCF-7, T47D cell lines	MCF-7: EqE_10_ = 0.02 µM, EqE_100_ = 0.08 µM T47D: EqE_10_ = 0.2 µM	[58]
	Belamphenone	In vitro	MCF-7, T47D cell lines	MCF-7: EqE_10_ = 0.8 µM, EqE_100_ = 12.8 µM T47D: EqE_10_ = 0.09 µM, EqE_100_ = 37.1 µM	[58]
	Tectorigenin	In vivo	Female Sprague Dawley rats	Stimulated luciferase production, cessation of pulsatile LH release, basal LH levels ↓, loss of BMD ↓, sustained uterine weight or estrogen-regulated uterine gene expression	[54]
Kidney protective activity					
	Belamcandaoid C	In vitro	Rat renal proximal tubular cells	TGF-β1-induced fibronectin expression ↓, inhibit the phosphorylation of Smad2/3	[17]
	Belamcandaoid M	In vitro	Rat renal proximal tubular cells	TGF-β1-induced fibronectin expression ↓, inhibit the phosphorylation of Smad2/3	[48]
	Belamchinane A	In vitro	D-gal-induced tubular cell	Inhibited cell senescence	
	Belamchinane B	In vitro	D-gal-induced tubular cell	Inhibited cell senescence	
	Belamchinane C	In vitro	D-gal-induced tubular cell	Inhibited cell senescence	[48]
	Belamchinane D	In vitro	D-gal-induced tubular cell	Inhibited cell senescence	[48]
Anti-complementary activity					
	BCP-A1	In vitro	6% sheep red blood cells	50% hemolytic inhibition concentrations: CH_50_ = 0.009 ± 0.003, AP_50_ = 0.015 ± 0.003	[13]
	BCP-B1	In vitro	6% sheep red blood cells	50% hemolytic inhibition concentrations: CH_50_ = 0.004 ± 0.001, AP_50_ = 0.028 ± 0.005	[13]
Anti-VSMC proliferation					
	Isoswertisin	In vitro	Vascular smooth muscle cells	VSMC proliferation ↓	[18]
	Embinin	In vitro	Vascular smooth muscle cells	VSMC proliferation ↓	[18]
	6″-*O*-Acetylembinin	In vitro	Vascular smooth muscle cells	VSMC proliferation ↓	[18]
	3″-*O*-Acetylembinin	In vitro	Vascular smooth muscle cells	VSMC proliferation ↓	[18]
	Iridin	In vitro	Vascular smooth muscle cells	VSMC proliferation ↓	[18]
Anti-tussive effect	*B. chinensis* extract	In vivo	18~22g healthy male ICR mice	Prolonged the latent period of cough induced by ammonia and decreased the cough times in 2min	[19]
Anti-psoriatic	Tectoridin	In vivo	SPF male C57BL/6 mice	TNF ↓, IL⁃6 ↓, IL⁃17A ↓, Th17/Treg ↓	[20]
Expectorant effect	*B. chinensis* extract	In vivo	18~22g healthy male ICR mice	The secretion of phenol red in trachea of mice ↑	[19]
Analgesic effect	*B. chinensis* extract	In vivo	18~22g healthy male ICR mice	The writhing times of mice ip 0.6% acetic acid solution ↓	[19]
Improve humoral immunity	*B. chinensis* extract	In vivo	18~22g healthy male ICR mice	Antibody hemolysin ↑	[19]
Inhibite PTP1b	Polycycloiridal L	In vitro	PTP1b	Inhibitory rate: 33.4%	[47]
	Polycycloiridal T	In vitro	PTP1b	Inhibitory rate: 32.9%	[47]

**Table 8:** The toxicity, extract, model and results of *B. chinensis* are summarized.

	Extracts/ Compounds	Type	Models/ Methods	Effects /Mechanisms	Refence
**Toxicity**	16-*O*-Acetylisoiridogermanal	In vivo	Killiefish (*Oryzias latipes*)	Median tolerance limit after 24 h: 3.5 µg/mL	[5]
	Belachinal	In vivo	Killiefish (*Oryzias latipes*)	Median tolerance limit after 24 h: 2.8 µg/mL	[5]
	Spiroiridal	In vivo	Killiefish (*Oryzias latipes*)	Median tolerance limit after 24 h: 1.6 µg/mL	[5]
	Dibelamcandal A	In vivo	*Pomacea canaliculata*	LC_50_ = 1.26 µg/mL, LC_95_ = 10.57 µg/mL	[50]
	*B. chinensis* methanol extract	In vivo	*Brine Shrimp nauplii*	LC_50_ = 16.218 µg/mL	[106]
	*B. chinensis* ethyl acetate extract	In vivo	*Brine Shrimp nauplii*	LC_50_ = 0.048 µg/mL	[106]
	*B. chinensis* ethanol extract	In vivo	Wistar rats	AST ↑, ALT ↑, TG ↓	[116]
	*B. chinensis* ethanol extract	In vivo	39 SPF Rats	spleen index ↓	[117]

**Table 9:** Proprietary Chinese medicines that include *B. chinensis*.

Proprietary Chinese Medicines	Traditional and Clinical Uses	Reference
Qing Yan Run Hou Wan	Clear away heat and throat issues, reduce swelling and relieve pain. Used for chest and diaphragm discomfort, thirst, irritability, cough with excessive phlegm, sore throat, and hoarseness of voice	[35]
Qing Ge Wan	Clears heat and reduces swelling and pain of the throat. Indicated for thirst and swollen and sore throat, hoarseness of voice, swollen cheeks, constipation with dry stools	[35]
Xiao Er Qing Fei Zhi Ke Pian	Clears heat, releases exterior, relieves cough, and resolves phlegm. For pediatric patients with fever, cough, restlessness, thirst, and dry stools caused by external wind-heat invasion and internal lung fire	[35]
Xiao Er Fei Re Ping Jiao Nang	Exerts antipyretic, expectorant, antitussive properties. Effective for pediatric pneumonia with phlegm-heat syndrome	[35]
Qing Yan Li Ge Wan	Demonstrates antipyretic, pharyngeal-soothing, anti-inflammatory, and analgesic properties. Effective for pharyngeal edema, facial flushing, excessive mucus, thoracic discomfort, bitter taste, xerostomia, constipation, and dark urine	[35]
She Ma Kou Fu Ye	Exhibits anti-inflammatory, expectorant, antitussive, and bronchodilatory effects. Effective for heat-transformed respiratory syndromes including productive cough with viscous sputum, chest tightness, wheezing, phlegm rales, fever presentation, red tongue body	[35]
Lu Si Ge Wan	Exerts lung-ventilating, phlegm-resolving, and antitussive actions. Clinically effective for pertussis and cough with phlegm obstruction syndrome	[35]
Xiao Er Yan Bian Ke Li	Demonstrates anti-inflammatory, analgesic, and detoxifying properties targeting upper respiratory tract inflammation. Effective for pediatric acute pharyngitis and tonsillitis	[35]
Gan Lu Xiao Du Wan	Aromatic dampness resolution and heat-clearing detoxification. Indicated for summerheat-dampness accumulation, presenting with fever, limb soreness, chest tightness, abdominal distension, and jaundice	[35]
Gui Lin Xi Gua Shuang	Clears heat, detoxifies, reduces swelling, alleviates pain. Indicated for chronic pharyngitis, tonsillitis, stomatitis, oral ulcers, and gingivitis	[35]
Jin Bei Tan Ke Qing Ke Li	Clears lung heat, arrests cough, resolves phlegm, and relieves wheezing. Indicated for cough with yellow sticky sputum and dyspnea due to phlegm-heat obstructing the lungs	[35]
Gan Mei Bing Ju Pian	Clears heat and restores voice. Hoarseness or aphonia due to wind-heat invading the lungs	[35]
Ke Gan Li Yan Kou Fu Ye	Dispels wind-heat, detoxifies, and soothes the throat. Indicated for fever with mild aversion to wind, headache, sore throat, nasal congestion with discharge, cough with sticky sputum, thirst and yellow nasal mucus	[35]

## Abbreviations

ABTS	2,2′-azino-bis-(3-ethylbenzenthiazoline-6-sulphonic) acids	Keap1	Kelch-like ECH associated protein 1
ABCA1	ATP binding cassette transporter A1	LDH	Lactate dehydrogenase
ABCG1	ATP binding cassette subfamily G Member 1	LDL-c	Low-density lipoprotein cholesterol
AChE	Acetylcholinesterase	LDLR	Low-density lipoprotein receptor
ACP5	Artrate resistant acid phosphatase 5	LTC4	Leukotriene C4
ALT	Alanine aminotransferase	LDL	Low-density lipoprotein
AOM	Azoxymethane	LPS	Lipopolysaccharide
AR	Androgen receptor	LXR	Liver X receptor
AST	Aspartate aminotransferase	MAPK	Mitogen-activated protein kinases
AUC	Area under the curve	MCP-1	Monocyte chemoattractant protein-1
BALF	Bronchoalveolar lavage fluid	MDA	Malondialdehyde
Bax	B-cell lymphoma-2-associated X protein	MIC	Minimum inhibitory concentration
BchE	Butyrylcholinesterase	MPP+	N-Methyl-4-Phenylpyridinium
Bcl-2	B-cell lymphoma-2	MRSA	Methicillin-resistant Staphylococcus aureus
bCTX	Beta *C*-terminated telopeptide of type I collagen	MMP1	Matrix metalloproteinase 1
BMD	Bone turnover, bone mineral density	MMP3	Matrix metalloproteinase 3
caspase-3	Cleaved cysteinyl aspartate specific proteinase-3	MMP9	Matrix metalloproteinase 9
CCl4	Carbon tetrachloride	NF-κB	Nuclear factor-κB
COX-2	Cyclooxygenase-2	NFATc1	Nuclear factor of activated T cells cytoplasmic 1
CPEI	Cytopathic effect inhibition assay	NIR	Near-infrared
CtsK	Cathepsin K	NO	Nitric oxide
DBIL	Direct bilirubin	Nrf2	Nuclear factor erythroid 2-related factor 2
DPPH	2,2-Diphenyl-1-picrylhydrazyl	OGD/R	Oxygen-glucose deprivation/reoxygenation
DSS	Extran sulfate sodium	OPN	Osteopontin
EC_50_	50% effective concentration	ovx	Ovariectomized
EMT	Epithelial-mesenchymal transition	Pam3CSK4	Bacterial lipoprotein
EPO	Erythropoietin	PCOS	Polycystic ovary syndrome
ER	Estrogen receptor	PCR	Polymerase chain reaction
ERK	Extracellular regulated protein kinases	PD	Parkinson’s disease
FBG	Fasting blood glucose	PDEF	Pleiotropic transcription factor
FRAP	Ferric ion reducing antioxidant power	PI3K	Phosphatidylinositol 3 kinase
GSH-Px	Glutathione peroxidase	PPAR γ	Peroxisome proliferator-activated receptor gamma
GSK-3β	Glycogen synthase kinase-3 beta	PR	Progesterone receptor
GSP	Glycosylated serum protein	PSA	Prostate specific antigen
HCE-2 cells	Human comeal epithelial cells-2	RANKL	Receptor activator of NF-κB ligand
Her-2	Human epidermal growth factor receptor 2	ROS	Reactive oxygen species
HIV	Human immunodeficiency virus	RAW 264.7	Mouse leukemia cells of monocyte macrophage
HMGCR	3-hydroxy-3-methylglutaryl-coenzyme A reductase	RT	RNA isolation and reverse transcriptase
HMOX1	Heme ooxygenase 1	SOD	Superoxide dismutase
HNE	Human neutrophil elastase	SLC3A2	Solute carrier family 3 member 2
IBIL	Indirect bilirubin	SORT1	Sortilin
IC_100_	Inhibitory concentration	SQLE	Squalene epoxidase
IC_50_	Half maximal inhibitory concentration	TC	Triglyceride
IFN-γ	Interferon-γ	TFR1	Transferrin receptor protein 1
IGF-1	Insulin like growth factor-1	TG	Total cholesterol
iNOS	Inducible nitric oxide synthase	Th17	T helper 17 cell
IL-10	Interleukin-10	TLm	Median tolerance limit
IL-1β	Interleukin-1β	TLR4	Toll-like receptor protein 4
IL-2	Interleukin-2	TNBC	Triple-negative breast cancer
IL-4	Interleukin-4	TNF	Tumor necrosis factor
IL-6	Interleukin-6	VC	Vitamin C
IL-12	Interleukin 12	VSMC	Vascular smooth muscle cell
